# Fast hospital discharge rates blur within-hospital ‘transmission footprint’ in bacterial genomes, as showcased with *Staphylococcus aureus*

**DOI:** 10.1371/journal.pcbi.1013982

**Published:** 2026-03-16

**Authors:** Sanni Översti, Mathilde Boumasmoud, Huldyrch F. Günthard, Hugo Sax, Annelies S. Zinkernagel, Roger D. Kouyos, Denise Kühnert

**Affiliations:** 1 Transmission, Infection, Diversification & Evolution Group, Max Planck Institute of Geoanthropology, Jena, Germany; 2 Max Planck Institute for Evolutionary Anthropology, Leipzig, Germany; 3 Department of Infectious Diseases and Hospital Epidemiology, University Hospital Zurich, Zurich, Switzerland; 4 Institute of Integrative Biology, Department of Environmental Systems Science, ETH Zürich, Zurich, Switzerland; 5 Institute of Medical Virology, University of Zurich, Zurich, Switzerland; 6 Department of Infectious Diseases, Bern University Hospital and University of Bern, Bern, Switzerland; 7 Center for Artificial Intelligence In Public Health Research, Robert Koch-Institute, Berlin, Germany; University of Washington, UNITED STATES OF AMERICA

## Abstract

The relatively slow mutation rates of bacterial pathogens impose severe limitations on phylodynamic analysis of bacterial outbreaks. However, whole-genome sequencing may enable accurate inference of bacterial transmission dynamics in health-care settings. We simulated the epidemic dynamics of a *Staphylococcus aureus* lineage using a stochastic model with a hospital and community compartment connected by patient admission and discharge. We generated synthetic genomic sequences and performed Bayesian phylodynamic inference on a proportion of samples from each simulated outbreak. When samples are obtained from both compartments, hospital transmission rate ($\lambda_{H}$) and community transmission rate ($\lambda_{C}$) are accurately estimated, if $\lambda_{H}$ is on the same scale as the discharge rate. If $\lambda_{H}$ is substantially lower than the discharge rate, a robust quantification of within-hospital transmission dynamics is challenging. Excluding samples from the community resulted in a notable underestimation of $\lambda_{H}$ when $\lambda_{H}\geq \lambda_{C}$. When transmission was ‘community-driven’, but sampling was restricted to hospital cases only, estimates are closer to the true $\lambda_{H}$, if hospital sampling proportion is known. Otherwise, $\lambda_{H}$ estimates reflected the transmission dynamics within the community. When using genomic data to estimate bacterial transmission rates in a health-care setting, it is essential to take into account the surrounding community. Many infections related to nosocomial outbreaks will not be observed within the hospital due to fast discharge rates. In the absence of usable genomic data from the community, alternative estimates of community transmission rates from publicly available data should be incorporated. Transmission rate estimates from nosocomial genomes alone need to be interpreted with care.

## Author summary

In our research, we explored how integrating evolutionary biology and epidemiology through a Bayesian phylodynamic inference method can improve our understanding and management of bacterial outbreaks. While this approach has been widely used for viruses, its application to bacterial pathogens, such as *Staphylococcus aureus*, has been more limited. *S. aureus* is a common human pathogen responsible for numerous infections in both community and healthcare settings, making it a significant public health concern globally.

Our simulation study aimed to assess the effectiveness of phylodynamic methods in investigating *S. aureus*-like outbreaks. We simulated various scenarios to evaluate how well phylodynamic analysis can track transmission dynamics within hospitals and surrounding communities. Our findings suggest that when bacterial genetic data from both hospital patients and the community are available, phylodynamics provides valuable insights into bacterial spread. However, the effectiveness of these methods diminishes significantly when only hospital data are considered. This highlights the importance of incorporating data from both sources to gain a comprehensive understanding of bacterial transmission in healthcare settings. Our study lays the groundwork for more effective strategies in monitoring and responding to bacterial outbreaks.

## Introduction

There is high potential in the analysis of genomic data from pathogen outbreaks [1], particularly in hospitals [2]. Whole genome sequencing (WGS) is proving to be an important tool for nosocomial outbreak management [3–8] and may help in choosing appropriate treatments to avoid selection of drug resistant pathogens [9]. Beyond applications in evolutionary medicine and hospital infection control [10,11], WGS data enable phylodynamic analysis of pathogen outbreaks. The field of phylodynamics makes use of the fact that pathogen evolution occurs at the same timescale as epidemiological events take place among hosts [12–15]. For example, phylodynamic analysis of WGS data allows robust estimation of the effective reproduction number and further epidemiological parameters from virus genome sequences [16–18]. Phylodynamic approaches can also infer the underlying host population structure, by introducing connected compartments, each governed by their own dynamics. For instance, in the context of HIV, this approach allowed to quantify the role of different transmission groups [19,20].

Whereas phylodynamics have provided insights into transmissibility, epidemiology and spatial dispersal of viruses, this approach has been less exploited for bacterial pathogens. Factors such as different disease dynamics (e.g., large numbers of asymptomatically colonised people) and relatively slow substitution rates, have been identified as possible challenges for bacterial phylodynamics [21]. Furthermore, as bacteria undergo frequent recombination events, they tend to have a complex genetic structure [22] that might not be correctly assessed with current phylodynamic models. Nevertheless, studies have shown that when whole-genome sequences are available, bacterial outbreaks can also be investigated using phylodynamics [23,24]. Moreover, when focusing on the core genome of a single lineage, bacterial phylogenies can display sufficient temporal signal within a phylodynamic framework [3].

Disentangling transmission in the hospital from that in its surrounding community is a case in point for both the importance and difficulty of phylodynamic inference of population structure for bacterial pathogens. On the one hand, it is essential to determine whether pathogens are acquired in the hospital or imported. On the other hand, quantifying the relative importance of transmission within each setting is challenging due to the fast discharge rate from the hospital to the community, and because many infections are caused by pathobionts, implying that only a small fraction of those colonized develop symptoms and may hence be detected by symptom-guided screening programs.

Here we assessed these challenges for *Staphylococcus aureus*, which is both a common commensal and major human pathogen, that causes infections in both healthy and immunocompromised individuals [25]. Its several lineages have distinct phenotypic features, such as virulence or drug-resistance, which impact transmission dynamics [26,27]. Alarmingly, *S. aureus* poses a notable risk of nosocomial transmission even in its drug sensitive form [28]. Some lineages have arisen in health-care settings and spread internationally within this ecological niche, while others have their transmission focus in the community [29–34]. Consequently, there has been a distinction between health care-associated methicillin-resistant (HA-MRSA) and community-associated methicillin-resistant (CA-MRSA) *S. aureus* lineages [35]. However, this distinction is blurring, as many lineages were reported to spread across community and hospitals [36]. In this simulation study, we assumed that we are tracking a single *S. aureus* lineage spreading at the interface of hospital and community and we investigated in a range of scenarios whether whole-genome data sampled from either both the hospital and the community or only from the hospital allow us to correctly estimate the corresponding transmission rates. As empirical datasets with a sufficient number of bacterial genomes that mirror these specific settings are currently unavailable, our study aims to fill this gap by using simulations to explore and represent plausible dynamics of *S. aureus* transmission. While this study centers on *S. aureus* focusing on simplified yet realistic transmission scenarios, its primary goal is to inform future empirical studies on effective sequencing strategies for *S. aureus*. Additionally, the aim is to encourage the exploration of transmission dynamics in other bacterial pathogens, such as *Pseudomonas spp.*, *Acinetobacter spp.*, and *Enterococci spp.* using similar simulation-based approaches that account for unique pathogen-specific evolutionary and epidemiological characteristics.

## Methods

### Bacterial infection dynamics

**Fig 1:**
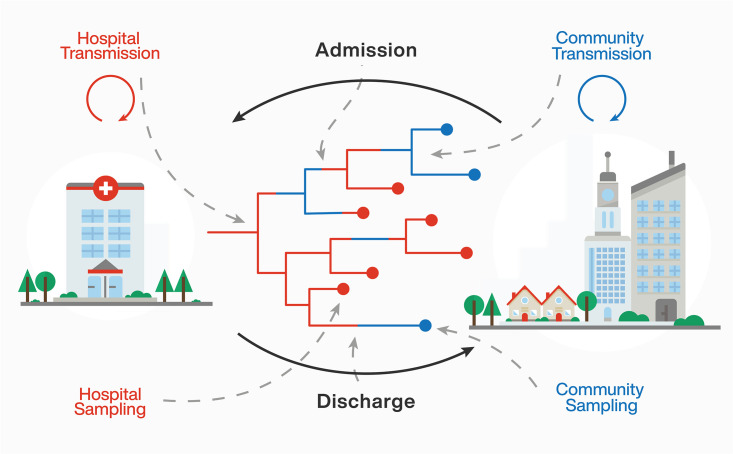
Schematic overview of transmission dynamics assumed in this study. The population is structured into two demes: ’hospital’ and ’community’, denoted with red and blue colours, respectively. Demes are connected through patient admission and discharge, illustrated with type-change events along the lineages of the transmission tree. Furthermore, transmission events and sampling events can occur within subpopulations. The sampled transmission tree is presented in the middle of the figure. For the corresponding full transmission tree, see S1 Fig.

The basis of the simulation study is a simple stochastic model of infection within two demes, the hospital *H* and its surrounding community *C*, that are connected by patient admission and discharge (Fig 1). New infections occur at transmission rates $\lambda_{H}$ in the hospital and $\lambda_{C}$ in the community. The migration rates between the two demes are based on known admission ($m_{CH}$) and discharge rates ($m_{HC}$) at the University Hospital Zurich: On average an individual is hospitalized once every 11.36 years, i.e., $m_{CH}=0.088y^{-1}$, and stays in the hospital for about 8.3 days, i.e., $m_{HC}$ = $44y^{-1}$[37]. The assumed discharge rate is consistent with the average hospital length of stay of 7.7 days reported across 36 OECD countries (Organisation for Economic Co-operation and Development; https://www.oecd.org/en/publications/health-at-a-glance-2023_7a7afb35-en/full-report/hospital-activity_7a57d1e4.html).

The rates defined here and in the following are exponentially distributed rates per year. This implies that the discharge rate, for example, is the inverse of the average period an individual stays in the hospital ($m_{HC}=\frac{1}{8.3\text{ days}}=0.1205d^{-1}$). That is, estimates of length of stay at the University Hospital Zurich were used to determine the discharge rate of the simulation model. With the bacterium *Staphylococcus aureus* in mind as a model organism, we assume for both demes a rate to become non-infectious $\delta =1y^{-1}$, which aligns with the median time to clearance represented in [38]. Here, recovery refers to the duration of *S. aureus* carriership. That is, an individual is colonized and able to transmit the pathogen for an average period of one year. While colonization generally refers to the existence of bacteria in the body without causing any illness, infection involves the invasion of the host’s bodily tissues by the pathogen that leads to disease. Here, we pool these groups into one “infected” group, as the pathogen can be transmitted both from colonized and infectious individuals. When infection causes symptoms, individuals may be diagnosed and sampled. Upon sampling patients are assumed to be treated and hence removed from the infectious pool.

The compartmental dynamics between hospital and community are given by the following stochastic events:


$$\;H\overset{\lambda_{H}}{⟶}\;2H\;\;\;\;\;\;\;\;\;\text{(transmission event within hospital)}\;C\overset{\lambda_{C}}{⟶}\;2C\;\;\;\;\;\;\;\;\text{(transmission event within community)}\;C\overset{m_{CH}}{⟶}\;H\;\;\;\;\;\;\;\;\text{(admission to hospital)}\;H\overset{m_{HC}}{⟶}\;C\;\;\;\;\;\;\;\;\text{(discharge from hospital)}\;H\overset{\mu_{H}}{⟶}\;R\;\;\;\;\;\;\;\;\;\;\text{(recovery event in hospital)}\;H\overset{\psi_{H}}{⟶}\;R\;\;\;\;\;\;\;\;\;\text{(sampling event in hospital)}\;H\overset{\mu_{C}}{⟶}\;R\;\;\;\;\;\;\;\;\;\;\text{(recovery event in community)}\;H\overset{\psi_{C}}{⟶}\;R\;\;\;\;\;\;\;\;\text{(sampling event in community)},$$ (1)

with $\mu_{C,H}$ and $\psi_{C,H}$ selected such that $\delta =\mu_{C}+\psi_{C}=\mu_{H}+\psi_{H}$, $s_{C}=\frac{\psi_{C}}{\delta }$ and $s_{H}=\frac{\psi_{H}}{\delta }$. Assuming an overall basic reproduction number of $R_{0}=1.2$, which is based on previous estimates [39], the transmission rates are chosen to reflect three main scenarios: (1) hospital-driven transmission rate, (2) equal transmission rates and (3) community-driven transmission rate.

For the hospital-driven transmission rate we assume three different scenarios with $\lambda_{H}=36.0\;y^{-1}$, $\lambda_{H}=45.0\;y^{-1}$ and $\lambda_{H}=49.5\;y^{-1}$. These values are chosen as the rate at which an infected individual is discharged from the hospital is 45 ($m_{HC}+\delta_{H}=44y^{-1}+1y^{-1}$) and thus chosen $\lambda_{H}$ values correspond to a single admission reproduction number ($R_{H}$) of slightly below, equal, or above one: i.e., 36/45 ($R_{H}=0.8$, below epidemic threshold), 45/45 ($R_{H}=1.0$) and 49.5/45 ($R_{H}=1.1$, above epidemic threshold). Here, the admission reproductive number corresponds to the average number of secondary infections produced by one infected case during a single hospital stay. Hence, it corresponds to the transmission rate $\lambda_{H}$ divided by the total rate of that case being removed from the hospital either by clearance (δ) or discharge ($m_{HC}$), hence $R_{H}=\frac{\lambda_{H}}{\delta +m_{HC}}$. The same applies for the community reproductive number ($R_{C}$); however, as δ ≫ $m_{CH}$, $R_{C}=\frac{\lambda_{C}}{\delta +m_{CH}}$ ≈ $\frac{\lambda_{C}}{\delta }$.

For the hospital transmission rates ($\lambda_{H}=36.0/45.0/49.5\;y^{-1}$) the corresponding within-community transmission rates $\lambda_{C}=1.0\;y^{-1}$, $\lambda_{C}=0.69\;y^{-1}$ and $\lambda_{C}=0.07\;y^{-1}$, respectively, are computed with next generation matrix [40] by assuming an overall reproduction number of $R_{0}=1.2$. For the equal transmission, both $\lambda_{H}$ and $\lambda_{C}$ are set to a value of 1.2, whereas for the community-driven transmission we assume transmission rates of $\lambda_{H}=0.75\;y^{-1}$ and $\lambda_{C}=1.2\;y^{-1}$. Details of the next generation matrix calculations are provided in S1 Text. Note that the hospital transmission rate becomes negligible in the next generation matrix calculations due to the high discharge rate.

For all scenarios, we performed simulations assuming a hospital sampling rate corresponding to a sampling proportion of 20% ($s_{H}=0.2$). In practice, this translates to every fifth hospital case being detected and sequenced. This proportion was arbitrarily selected, as it falls within a realistically achievable range, particularly in high-income countries. Sensitivity analyses with hospital sampling proportions of 5% and 10% were also performed. For the community, three different sampling rates were assumed, corresponding to community sampling proportions of $s_{C}=0.01$, $s_{C}=0.001$ and $s_{C}=0.0001$. The same admission and discharge rates ($m_{HC}$ and $m_{CH}$, respectively) are used in all scenarios.

Simulations are thus performed under the following scenarios:


$$\delta_{C}=\delta_{H}=1.0\;y^{-1}$$

$$m_{HC}=44.0\;y^{-1}\text{, }m_{CH}=0.088\;y^{-1}$$

$$s_{C}=0.01\text{ / }s_{C}=0.001\text{ / }s_{C}=0.0001$$

$$s_{H}=0.2$$

1. Hospital-driven transmission (HDT) rate:
  (a) $\lambda_{C}=1.0y^{-1}\text{, }\lambda_{H}=36y^{-1}$
  (b) $\lambda_{C}=0.69y^{-1}\text{, }\lambda_{H}=45y^{-1}$
  (c) $\lambda_{C}=0.07y^{-1}\text{, }\lambda_{H}=49.5y^{-1}$
2. Equal transmission (ET) rates:
  $$\lambda_{C}=1.2y^{-1}\text{, }\lambda_{H}=1.2y^{-1}$$
3. Community-driven transmission (CDT) rate:
  $$\lambda_{C}=1.2y^{-1}\text{, }\lambda_{H}=0.75y^{-1}$$

Table 1 summarizes the key parameter differences between the simulation scenarios. For each scenario, we simulated 100 nosocomial outbreak genomes by producing transmission trees using MASTER [41]. Entire transmission chains were simulated, and from each replicate a subsample was taken by allowing 1.0%, 0.1% or 0.01% of the infected population to be sampled in the community, and 20% in the hospital. Each simulation started with a single infected individual in the community compartment, which constitutes the root of the full transmission tree. After reaching 100 samples, the full tree is pruned to only contain the sampled individuals. For all scenarios, the sampling process started simultaneously with the transmission process.

**Table 1:** Key differences in phylodynamic model assumptions across simulation scenarios. Scenario abbreviations are as follows: HDT = hospital-driven transmission, ET = equal transmission, and CDT = community-driven transmission. All scenarios were simulated using the same values for the rate of becoming non-infectious ($\delta =1.0y^{-1}$), hospital admission rate ($m_{CH}=0.088y^{-1}$), and discharge rate ($m_{HC}=44.0y^{-1}$). For phylodynamic inference, either a two-deme model (*bdmm*) or a one-deme model (*bdsky*) was applied. Genomic evolution in all scenarios was simulated using the HKY substitution model with *κ* = 4.04 and nucleotide frequencies A: 0.34, C: 0.16, G: 0.16, and T: 0.34. For each scenario, parameters of the sequence evolution model were inferred using default prior distributions during Bayesian analysis. Results for the main scenarios are provided in Tables 2, 3, 4, 5. Results for HDT (a) sensitivity 1 and 2 are provided in S1 Table.

Scenario	Simulated value*s* (*λ_C,H_* in unit*s* of *y*^−1^)	Phylodynamic model for the inference	Estimated parameters	Fixed parameters
HDT (a)	*λ_C_* = 1.0, *λ_H_* = 36.0 *s_C_* = 0.01/0.001/0.0001 *s_H_* = 0.2	*bdmm*	*λ_C_*, *λ_H_*, *δ*	*s_C_*, *s_H_*, *m_CH_*, *m_HC_*
HDT (a)’	*λ_C_* = 1.0, *λ_H_* = 36.0 *s_C_* = 0.0 *s_H_* = 0.2	*bdsky*	*λ* *_H_*	*s_H_*, *δ*
HDT (a) sensitivity 1	*λ_C_* = 1.0, *λ_H_* = 36.0 *s_C_* = 0.001 *s_H_* = 0.1	*bdmm*	*λ_C_*, *λ_H_*, *δ*	*s_C_*, *s_H_*, *m_CH_*, *m_HC_*
HDT (a) sensitivity 2	*λ_C_* = 1.0, *λ_H_* = 36.0 *s_C_* = 0.001 *s_H_* = 0.05	*bdmm*	*λ_C_*, *λ_H_*, *δ*	*s_C_*, *s_H_*, *m_CH_*, *m_HC_*
HDT (b)	*λ_C_* = 0.69, *λ_H_* = 45.0 *s_C_* = 0.01/0.001/0.0001 *s_H_* = 0.2	*bdmm*	*λ_C_*, *λ_H_*, *δ*	*s_C_*, *s_H_*, *m_CH_*, *m_HC_*
HDT (c)	*λ_C_* = 1.2, *λ_H_* = 49.5 *s_C_* = 0.01/0.001/0.0001 *s_H_* = 0.2	*bdmm*	*λ_C_*, *λ_H_*, *δ*	*s_C_*, *s_H_*, *m_CH_*, *m_HC_*
ET	*λ_C_* = 1.2, *λ_H_* = 1.2 *s_C_* = 0.01/0.001/0.0001 *s_H_* = 0.2	*bdmm*	*λ_C_*, *λ_H_*, *δ*	*s_C_*, *s_H_*, *m_CH_*, *m_HC_*
ET’	*λ_C_* = 1.2, *λ_H_* = 1.2 *s_C_* = 0.0 *s_H_* = 0.2	*bdsky*	*λ* *_H_*	*s_H_*, *δ*
CDT	*λ_C_* = 1.2, *λ_H_* = 0.75 *s_C_* = 0.01/0.001/0.0001 *s_H_* = 0.2	*bdmm*	*λ_C_*, *λ_H_*, *δ*	*s_C_*, *s_H_*, *m_CH_*, *m_HC_*
CDT’	*λ_C_* = 1.2, *λ_H_* = 0.75 *s_C_* = 0.0 *s_H_* = 0.2	*bdsky*	*λ* *_H_*	*s_H_*, *δ*
CDT”	*λ_C_* = 1.2, *λ*H = 0.75 *s_C_* = 0.0 *s*_*H*_ = 0.2	*bdsky*	*λ_H_*, *s_H_*	*δ*

**Fig 2:**
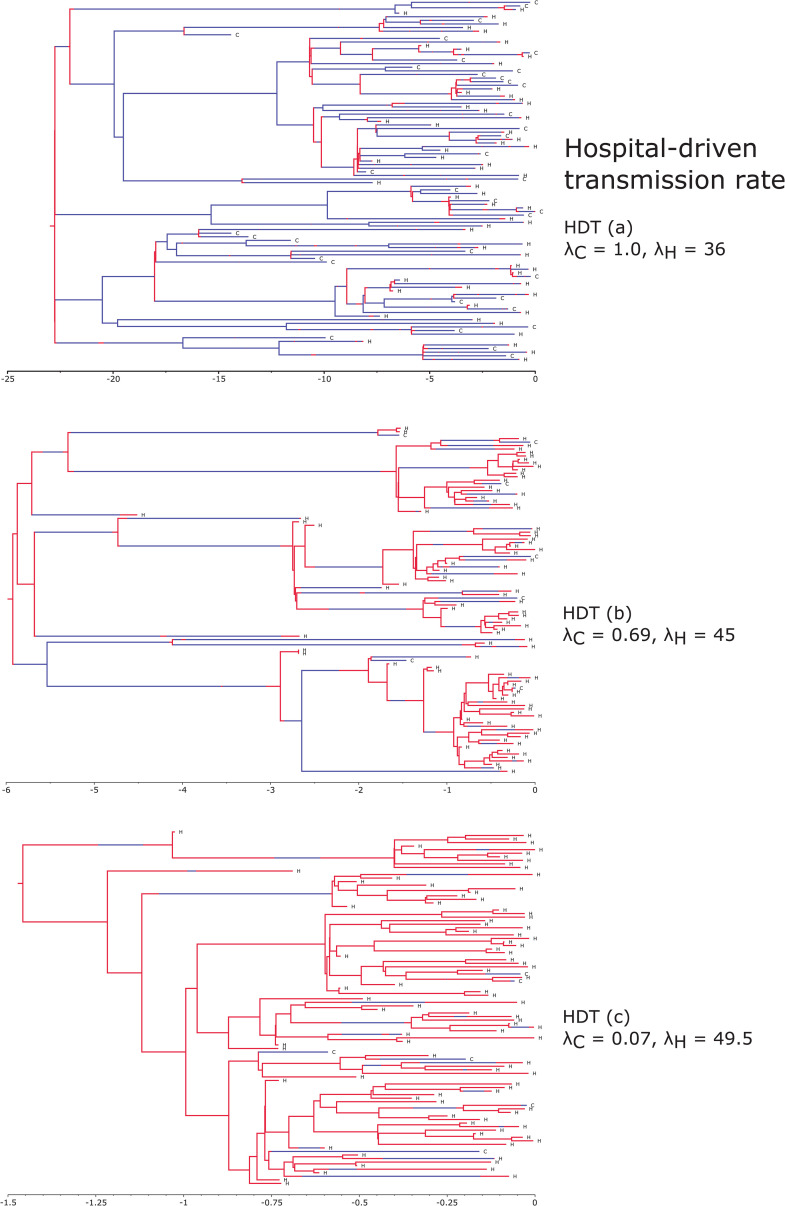
Examples of sampled transmission trees simulated based on hospital-driven transmission rates (scenarios HDT (a)–(c)). Branch colors indicate the community (blue) and hospital (red). Tip labels denote the deme from which the sample was collected, with ’C’ indicating community and ’H’ indicating hospital. In all panels, the x-axis represents time, here with the unit being years. All trees shown were generated under the assumption that 0.1% of infected individuals from the community and 20% from the hospital were sampled.

**Fig 3:**
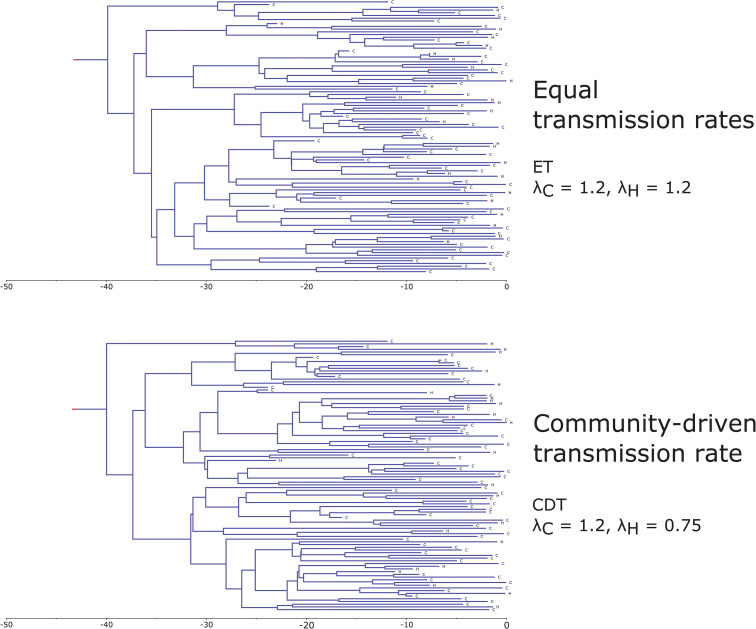
Examples of sampled transmission trees simulated based on equal and community-based transmission rates (scenarios ET and CDT, respectively). Branch colors indicate the community (blue) and hospital (red). Tip labels denote the deme from which the sample was collected, with ‘C’ indicating community and ‘H’ indicating hospital. In both panels, the x-axis represents time, here with the unit being years. Both trees shown were generated under the assumption that 0.1% of infected individuals from the community and 20% from the hospital were sampled.

To illustrate the underlying transmission processes and highlight the key differences between the simulation scenarios, we present examples of simulated transmission trees in Figs 2 and 3. For each main scenario, these sampled transmission trees were generated assuming a community sampling rate of $s_{C}$=0.001. Fig 2 showcases simulated transmission trees based on a hospital-driven transmission rate (scenarios HDT (a) – (c)), while Fig 3 provides examples of equal transmission and community-driven transmission trees (scenarios ET and CDT, respectively). In both figures, each divergence in the tree represents a transmission event, and the branches of the trees are colored according to the demes: blue for community and red for hospital. Additionally, the tip labels indicate the deme from which the sample was obtained, with ‘C’ representing community and ‘H’ representing hospital. In the example hospital-driven transmission trees, the number of samples obtained from the community, as well as the number of transmission events occurring within the community, decreases as the ratio of $\lambda_{H}$ to the rate at which an infected individual is discharged from the hospital increases (Fig 2). In contrast, the sampled transmission trees generated under equal and community-driven transmission rates show a similar pattern, with the majority of samples being acquired from the community (Fig 3). Additionally, in both of these example trees, all transmission events occur within the community. While these trees are intended to illustrate the key differences between scenarios, it is important to note that due to the stochastic nature of the underlying processes, transmission trees simulated under the same epidemiological parameters can exhibit significant variation.

Additionally, we considered a special case in which genomic data is available only from the hospital. For this purpose, an additional set of transmission trees was simulated, as described above, under a model assuming stochastic transmission dynamics between the hospital and the surrounding community. However, bacterial sequences were assumed to be retrieved only from the hospital and thus the community sampling rate was set to 0. In this scenario we perform inference with an intentional model misspecification, inferring parameters from a 1-deme model, as it is likely that researchers would employ a 1-deme model if they only had data from one deme. These additional analyses were performed for scenarios HDT (a), ET, and CDT and their results are labelled as HDT (a)’, ET’, and CDT’, respectively.

Subsequently, for all scenarios we simulated nucleotide sequences with a length of 2.6 Mb, mimicking the cumulative length of the *Staphylococcus aureus* core genes [3]. Sequences along the phylogenies were simulated with MASTER that builds on SeqGen simulation tool [42]. Genome evolution along the transmission tree branches was modelled according to [43] and [44] by using a HKY substitution model (transition/transversion ratio κ=4.04 and nucleotide frequencies set to A:0.34, C:0.16, G:0.16, T:0.34) with gamma distributed rate heterogeneity (with four gamma categories and shape parameter set to 1.0). Further, a strict clock model with clock rate $\mu =1.6\times 10^{-6}$ substitutions/site/year, estimated based on [45], was used.

### Bayesian inference of transmission rates

To test if the true transmission rates from the simulated outbreaks can be recovered, the resulting simulated genomic sequences are analyzed in the Bayesian MCMC framework BEAST2 v2.6.2 [46]. The BEAST2 package *bdmm* [47] allows inference under the two-compartment model described above.

The transmission dynamics are inferred through transmission rates within community ($\lambda_{C}$) and hospital ($\lambda_{H}$) together with rate to become non-infectious (*δ*), which are reconstructed jointly with the phylogenies. In addition, we inferred the transmission dynamics through the parametrization using the community and admission reproduction numbers, $R_{C}=\frac{\lambda_{C}}{\delta +m_{CH}}$ and $R_{H}=\frac{\lambda_{H}}{\delta +m_{HC}}$, respectively. As a prior distribution a uniform distribution (0, 100) was assumed for above mentioned parameters. To avoid potential model nonidentifiability arising from correlations between the within-compartment rates of each deme [48], all remaining epidemiological parameters, i.e., the sampling proportion as well as the admission and discharge rates, were set to their true values during the phylodynamic reconstruction, unless stated otherwise (i.e., scenario CDT”, see below).

The Bayesian phylodynamic inference was conducted using a HKY substitution model with gamma-distributed rate heterogeneity by accounting for a proportion of invariant sites. Parameters related to the substitution model were estimated under default prior distributions. A strict molecular clock was applied, with the rate fixed to the true value used in the simulations.

For scenarios in which only hospital cases were sampled (i.e., HDT (a)’, ET’, and CDT’), the *bdsky* model [48] was used, which is a one-dimensional special case of the *bdmm* model. Similarly to the scenarios mentioned above, the hospital sampling proportion was fixed to its true value ($s_{H}=0.2$). Moreover, for the community-driven transmission scenario CDT’ we further tested a sub-scenario CDT” where $s_{H}$ was estimated. For all scenarios assuming hospital sampling only, the rate to become non-infectious was fixed to its true value ($\delta =1.0y^{-1}$), in order to test the impact of and accuracy in estimating $s_{H}$ instead. Differences between the simulation scenarios for Bayesian inference are highlighted in Table 1.

In both models, *bdmm* and *bdsky*, the genomic tree is assumed to be a good approximation of the transmission tree [49]. Depending on the scenario, for each replicate we run the MCMC chain in BEAST2 for 10,000,000–30,000,000 steps to obtain effective sample size (ESS) of 200 or higher for each parameter included in the model (see S2 Text). Simulation files are provided in https://github.com/tidelab/S.aureus_hospital_outbreak.

Lastly, to further compare the transmission dynamics between community and hospital settings, we computed the posterior odds ratio (OR) for scenarios assuming samples were taken from both demes, defined as the ratio of the number of posterior samples where $\lambda_{C}<\lambda_{H}$ to those where $\lambda_{C}>\lambda_{H}$. Additionally, the ratio $\lambda_{C}/\lambda_{H}$ was computed for each sample. For each scenario, considering only replicates with ESS>200 for all model parameters, we report the following summary statistics: the median posterior odds, interquartile range (IQR) of posterior OR, median $\lambda_{C}/\lambda_{H}$, IQR of $\lambda_{C}/\lambda_{H}$, and the proportion of replicates in which posterior odds exceeded 1.

## Results

The aim of this study was to utilize simulated *Staphylococcus aureus* outbreaks in a hospital environment to investigate whether bacterial genomes from nosocomial outbreaks can be used to infer the transmission dynamics of bacterial pathogens in health-care settings. Stochastic transmission dynamics were simulated under three main scenarios by assuming hospital-driven, equal or community-driven transmission rates. Using simulated datasets, we investigated how well a two-deme (hospital and community) or one-deme (hospital-only) birth-death model can recover epidemiological quantities. Model performance was assessed by evaluating accuracy and precision.

**Fig 4:**
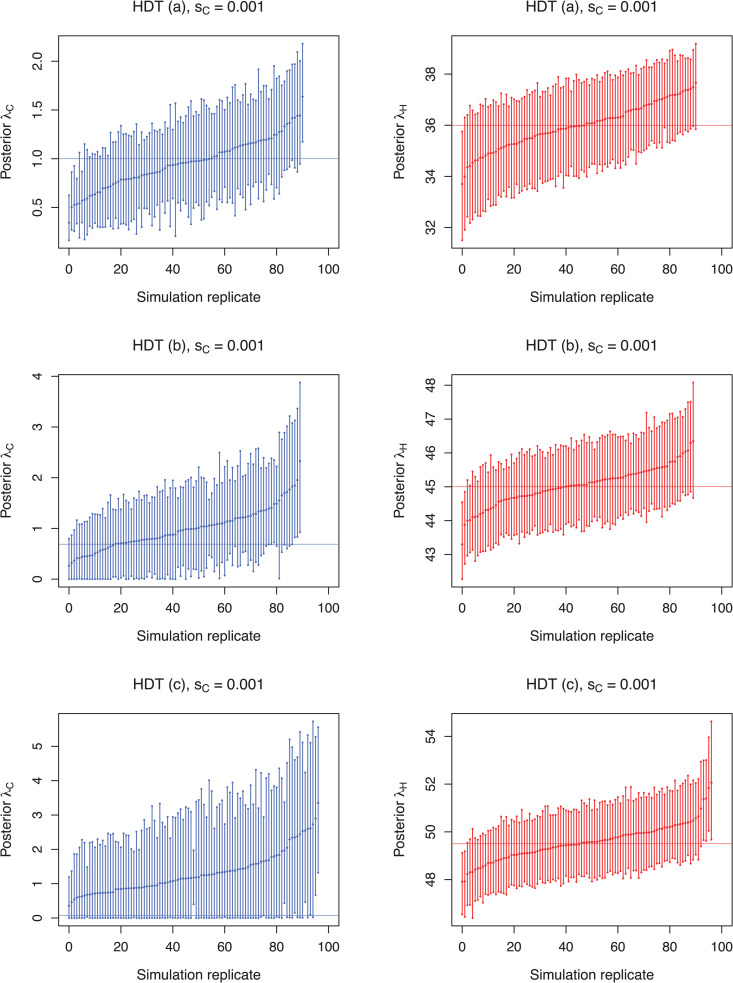
Estimated transmission rates for scenarios simulated under the assumption of hospital-driven transmission (HDT) and a community sampling proportion of *S*_*C*_ = 0.001. In scenarios HDT **(a)**, HDT **(b)**, and HDT (c) the hospital transmission rates were assumed to be lower, equal, or higher, respectively, than the assumed rate at which an infected individual is discharged from the hospital (i.e., $m_{HC}$ + $\delta_{H}$ = $44y^{-1}$ + $1y^{-1}$ = 45). Each bar represents the 95% HPD interval from a simulation replicate with a point denoting the median estimate. Horizontal lines indicate the true transmission rates within the community (blue) and the hospital (red). For clarity, simulation replicates are displayed in ascending order based on their mean values.

The Bayesian posterior estimates inferred from each scenario are summarized in Tables 2, 3 and 4 for sampling proportions of $s_{C}=0.01$, $s_{C}=0.001$ and $s_{C}=0.0001$, respectively. In the hospital-driven scenarios the true transmission rate within the hospital is either identical (i.e., HDT (b)) or relatively close (HDT (a) and HDT (c)) to the discharge rate and hence much higher than the transmission rate in the community. In these cases, hospital transmission rates are recovered well from the simulated sequences (Fig 4 for $s_{C}=0.001$, see S2 and S3 Figs for sampling schemes $s_{C}=0.01$ and $s_{C}=0.0001$). Furthermore, the community transmission rate is inferred well, particularly when the hospital transmission rate is somewhat lower than the discharge rate (Scenario HDT (a) where $m_{HC}=44y^{-1}$ > $\lambda_{H}=36.0\;y^{-1}$). Whereas no notable differences arise in hospital transmission rate accuracy and precision between different sampling schemes, for $\lambda_{C}$ the highest precision is obtained with the highest community sampling rate of 1% ($s_{C}=0.01$).To further evaluate the impact of hospital sampling rate on the inferred results under the hospital-driven transmission scenario, we performed additional sensitivity analyses for scenario HDT (a), assuming hospital sampling proportions of 10% and 5%. As shown in S1 Table, the parameters of interest were inferred relatively robustly across all community sampling schemes, even at the lower hospital sampling rates of $s_{H}=0.1$ and $s_{H}=0.05$. However, the number of simulation replicates achieving the requested ESS>200 decreased at these lower sampling rates, particularly for scenarios with $s_{C}=0.001$ and $s_{C}=0.0001$, despite adjustments made to certain operators to improve MCMC efficiency (S2 Text).

**Table 2:** Summary of the Bayesian posterior inference results from each simulation scenario assuming a community sampling rate of $s_{C}=0.01$. Scenario abbreviations are as follows: HDT = hospital-driven transmission, ET = equal transmission, and CDT = community-driven transmission. The column labelled ESS>200 indicates the number of replicates (out of 100) in which all parameters had an effective sample size (ESS) of at least 200. Epidemiological parameters not listed in the third column (e.g., admission and discharge rates as well as sampling proportions) were fixed to their true values. The ‘Relative error‘ column reflects the average relative absolute deviation, calculated as |median−truth|/truth. The ‘Relative bias‘ column shows the average relative deviation of the median estimate from the true value, calculated as (median−truth)/truth. Relative 95% highest posterior density (HPD) widths are computed as (upperbound – lowerbound)/truth. The ‘95% HPD accuracy‘ column indicates the number of replicates in which the 95% HPD interval included the true value for each parameter.

Scenario	ESS>200	Parameter	Truth	Median	Relative error	Relative bias	Relative HPD width	95% HPD accuracy
HDT (a)	93							
		$\lambda_{C}$	1.00	0.99	0.12	-0.01	0.71	97.00
		$\lambda_{H}$	36.00	35.79	0.03	-0.01	0.15	98.00
		*δ*	1.00	0.98	0.11	-0.02	0.66	98.00
HDT (b)	88							
		$\lambda_{C}$	0.69	0.89	0.48	0.29	2.20	92.00
		$\lambda_{H}$	45.00	44.96	0.01	0.00	0.07	93.00
		*δ*	1.00	1.10	0.24	0.10	1.15	93.00
HDT (c)	96							
		$\lambda_{C}$	0.07	1.00	13.31	13.31	32.83	88.00
		$\lambda_{H}$	49.50	49.65	0.01	0.00	0.07	95.00
		*δ*	1.00	1.13	0.26	0.13	1.17	96.00
ET	100							
		$\lambda_{C}$	1.20	1.16	0.07	-0.03	0.32	91.00
		$\lambda_{H}$	1.20	9.38	6.82	6.82	16.44	93.00
		*δ*	1.00	0.99	0.09	-0.01	0.40	93.00
CDT	100							
		$\lambda_{C}$	1.20	1.16	0.08	-0.04	0.32	85.00
		$\lambda_{H}$	0.75	9.26	11.34	11.34	26.17	89.00
		*δ*	1.00	0.98	0.10	-0.02	0.40	88.00

**Table 3:** Summary of the Bayesian posterior inference results from each simulation scenario assuming a community sampling rate of $s_{C}=0.001$. Scenario abbreviations are as follows: HDT = hospital-driven transmission, ET = equal transmission, and CDT = community-driven transmission. The column labelled ESS>200 indicates the number of replicates (out of 100) in which all parameters had an effective sample size (ESS) of at least 200. Epidemiological parameters not listed in the third column (e.g., admission and discharge rates as well as sampling proportions) were fixed to their true values. The ‘Relative error‘ column reflects the average relative absolute deviation, calculated as |median−truth|/truth. The ‘Relative bias‘ column shows the average relative deviation of the median estimate from the true value, calculated as (median−truth)/truth. Relative 95% highest posterior density (HPD) widths are computed as (upper bound−lower bound)/truth. The ‘95% HPD accuracy’ column indicates the number of replicates in which the 95% HPD interval included the true value for each parameter.

Scenario	ESS>200	Parameter	Truth	Median	Relative error	Relative bias	Relative HPD width	95% HPD accuracy
HDT (a)	91							
		$\lambda_{C}$	1.00	0.96	0.21	-0.04	0.94	93.00
		$\lambda_{H}$	36.00	35.98	0.02	0.00	0.10	99.00
		*δ*	1.00	0.96	0.19	-0.05	0.85	90.00
HDT (b)	90							
		$\lambda_{C}$	0.69	0.99	0.55	0.44	2.45	96.00
		$\lambda_{H}$	45.00	45.03	0.01	0.00	0.05	98.00
		*δ*	1.00	1.15	0.23	0.15	1.12	96.00
HDT (c)	97							
		$\lambda_{C}$	0.07	1.31	17.75	17.75	44.92	92.00
		$\lambda_{H}$	49.50	49.58	0.01	0.00	0.06	94.00
		*δ*	1.00	1.12	0.25	0.12	1.13	94.00
ET	100							
		$\lambda_{C}$	1.20	1.14	0.10	-0.05	0.38	92.00
		$\lambda_{H}$	1.20	6.15	4.13	4.13	11.79	96.00
		*δ*	1.00	0.95	0.12	-0.05	0.48	93.00
CDT	100							
		$\lambda_{C}$	1.20	1.12	0.09	-0.06	0.38	92.00
		$\lambda_{H}$	0.75	6.16	7.21	7.21	19.00	93.00
		*δ*	1.00	0.93	0.11	-0.07	0.48	93.00

**Table 4:** Summary of the Bayesian posterior inference results from each simulation scenario assuming a community sampling rate of $s_{C}=0.0001$. Scenario abbreviations are as follows: HDT = hospital-driven transmission, ET = equal transmission, and CDT = community-driven transmission. The column labelled ESS>200 indicates the number of replicates (out of 100) in which all parameters had an effective sample size (ESS) of at least 200. Epidemiological parameters not listed in the third column (e.g., admission and discharge rates as well as sampling proportions) were fixed to their true values. The ‘Relative error‘ column reflects the average relative absolute deviation, calculated as |median−truth|/truth. The ‘Relative bias‘ column shows the average relative deviation of the median estimate from the true value, calculated as (median−truth)/truth. Relative 95% highest posterior density (HPD) widths are computed as (upper bound−lower bound)/truth. The ‘95% HPD accuracy‘ column indicates the number of replicates in which the 95% HPD interval included the true value for each parameter. For scenario HDT (b) replicates 3136 and 3189 as well as for scenario HDT (c) replicates 3112 and 3152 were excluded (for details, see S3 Text).

Scenario	ESS>200	Parameter	Truth	Median	Relative error	Relative bias	Relative HPD width	95% HPD accuracy
HDT (a)	86							
		$\lambda_{C}$	1.00	0.98	0.21	-0.02	1.07	95.00
		$\lambda_{H}$	36.00	35.90	0.02	0.00	0.10	98.00
		*δ*	1.00	0.97	0.20	-0.03	0.96	92.00
HDT (b)	89							
		$\lambda_{C}$	0.69	1.00	0.57	0.45	2.54	92.00
		$\lambda_{H}$	45.00	44.97	0.01	0.00	0.05	97.00
		*δ*	1.00	1.11	0.21	0.11	1.10	97.00
HDT (c)	97							
		$\lambda_{C}$	0.07	1.92	26.38	26.38	60.68	86.00
		$\lambda_{H}$	49.50	49.54	0.01	0.00	0.06	98.00
		*δ*	1.00	1.20	0.27	0.20	1.28	94.00
ET	99							
		$\lambda_{C}$	1.20	1.07	0.13	-0.11	0.46	84.00
		$\lambda_{H}$	1.20	6.90	4.75	4.75	12.36	92.00
		*δ*	1.00	0.88	0.16	-0.12	0.57	85.00
CDT	100							
		$\lambda_{C}$	1.20	1.07	0.13	-0.11	0.45	82.00
		$\lambda_{H}$	0.75	6.41	7.55	7.55	19.66	94.00
		*δ*	1.00	0.87	0.16	-0.13	0.56	83.00

**Fig 5:**
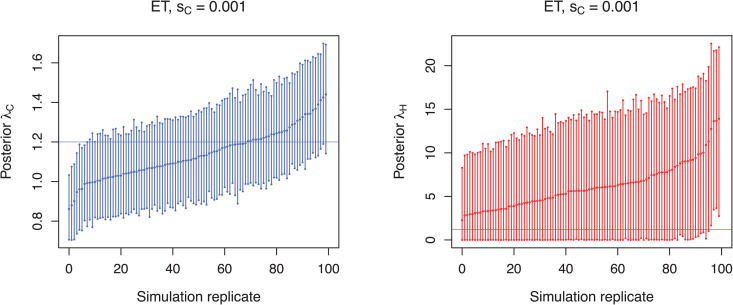
Estimated transmission rates for scenario simulated under the assumption of equal transmission (ET) and a community sampling proportion of *s_C_* = 0.001. Each bar represents the 95% HPD interval from a simulation replicate with a point denoting the median estimate. Horizontal lines indicate the true transmission rates within the community (blue) and the hospital (red). For clarity, simulation replicates are displayed in ascending order based on their mean values.

In the equal transmission scenario the community transmission rate is estimated well, but the transmission rate for the hospital is not (Fig 5 for $s_{C}=0.001$, see S4 Fig for other sampling schemes). Although inferred hospital transmission rates are estimated accurately (95% HPD accuracy: 92–96%), the 95% highest posterior density (HPD) intervals are large and estimates are heavily biased towards higher transmission rates (relative bias 4.1–6.8). In fact, with an average of 6.2–9.4 the estimated median hospital transmission rates are higher than the estimated median community transmission rates (average 1.1–1.2 for all sampling schemes). However, the qualitative regime is inferred correctly when considering that the admission reproductive number $R_{H}$ is well below one (S6–S8 Figs, see also below). This is further supported by the finding that, across all community sampling rates, the HDT scenarios consistently produced extremely high posterior odds ratios (median = 1000), indicating strong support for lower transmission rates in the community compared to hospital settings (S2 Table). Correspondingly, the median $\lambda_{C}/\lambda_{H}$ ratios for HDT scenarios remained low (0.02–0.04). In contrast, the ET and CDT scenarios produced moderate posterior odds (8–13), and higher $\lambda_{C}/\lambda_{H}$ ratios (0.55–0.97), reflecting more balanced or community-skewed transmission dynamics. The proportion of replicates with posterior odds >1 was close to or equal to 1 in all scenarios, indicating strong or consistent directional evidence across replicates.

**Fig 6:**
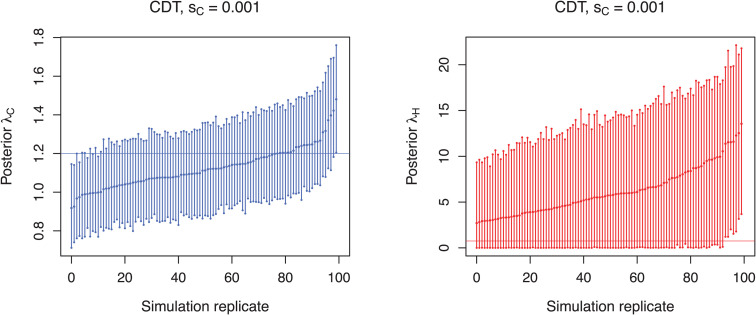
Estimated transmission rates for scenario simulated under the assumption of community-driven transmission (CDT) and a community sampling proportion of *s_C_* = 0.001. Each bar represents the 95% HPD interval from a simulation replicate with a point denoting the median estimate. Horizontal lines indicate the true transmission rates within the community (blue) and the hospital (red). For clarity, simulation replicates are displayed in ascending order based on their mean values.

In scenario CDT the community transmission rate is estimated well, but the estimation of the lower hospital transmission rate is highly biased (Fig 6 for $s_{C}=0.001$, see S5 Fig for other sampling schemes). Again, the median hospital transmission rate is higher than the median community transmission rate in all simulation replicates for each sampling scheme.

**Fig 7:**
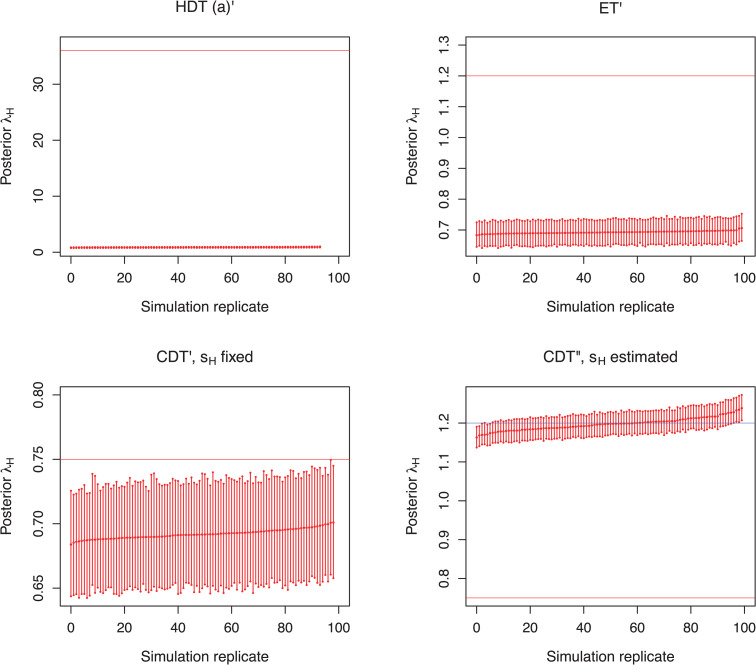
Estimated transmission rates for scenarios simulated under the assumption of sampling hospital cases only, i.e., scenarios HDT (a)’, ET, CDT’, and CDT”. In scenario CDT’ for the inference the hospital sampling proportion was fixed to its true value ($s_{H}=0.2$), whereas in scenario CDT” $s_{H}$ was estimated. Each bar represents the 95% HPD interval from a simulation replicate with a point denoting the median estimate. Horizontal lines represent the true transmission rates. In scenario CDT”, the estimated hospital transmission rate aligns more closely with the true community rate (blue line) rather than the actual hospital rate (red line). For clarity, simulation replicates are displayed in ascending order based on their mean values.

When only taking samples from within the hospital for scenarios HDT (a)’ and ET’ the hospital transmission rate is substantially underestimated (Table 5 and Fig 7). When limiting the sampling process to hospitalized patients under the community-driven transmission, $\lambda_{H}$ is similarly underestimated, if the sampling proportion $s_{H}$ within the hospital is known (scenario CDT’, Fig 7). Median estimates are, however, closer to the true value than in corresponding scenarios simulated under hospital-driven or equal transmissions (Table 5). When the within hospital sampling proportion is unknown (scenario CDT”), the estimated transmission rate represents what happens in the community and the sampling proportion is heavily underestimated (Table 5 and Fig 7).

**Table 5:** Summary of the Bayesian posterior inference results from scenarios, where samples were obtained exclusively from the hospital. Scenario abbreviations are as follows: HDT = hospital-driven transmission, ET = equal transmission, and CDT = community-driven transmission. The column labelled ESS>200 indicates the number of replicates (out of 100) in which all parameters had an effective sample size (ESS) of at least 200. Epidemiological parameters not listed in the third column (e.g., admission and discharge rates) were fixed to their true values. Sampling proportions were fixed to their true values in all scenarios except CDT”. The ‘Relative error‘ column reflects the average relative absolute deviation, calculated as |median−truth|/truth. The ‘Relative bias’ column shows the average relative deviation of the median estimate from the true value, calculated as (median−truth)/truth. Relative 95% highest posterior density (HPD) widths are computed as (upper bound−lower bound)/truth. The ‘95% HPD accuracy’ column indicates the number of replicates in which the 95% HPD interval included the true value for each parameter.

Scenario	ESS>200	Parameter	Truth	Median	Relative error	Relative bias	Relative HPD width	95% HPD accuracy
HDT (a)’	94							
		$\lambda_{H}$	36.00	0.87	0.98	-0.98	4e−3	0.00
ET’	100							
		$\lambda_{H}$	1.20	0.69	0.42	-0.42	0.07	0.00
CDT’	99							
		$\lambda_{H}$	0.75	0.69	0.08	0.08	0.11	0.00
CDT”	100							
		$\lambda_{H}$	0.75	1.20	0.60	0.60	0.08	0.00
		$s_{H}$	0.20	4e−4	1.00	-1.00	2e−3	0.00

In addition to transmission rates, we also assessed the accuracy of the inferred community and admission reproduction numbers, $R_{C}=\frac{\lambda_{C}}{\delta +m_{CH}}$ and $R_{H}=\frac{\lambda_{H}}{\delta +m_{HC}}$, respectively. When community and admission reproductive numbers are nearly equal (scenarios HDT (a) and HDT (b)), both quantities are relatively well estimated (S6–S8 Figs). However, when $R_{H}$ is substantially higher than $R_{C}$ (scenario HDT (c), only $R_{H}$ is being inferred accurately. On the contrary, for scenarios ET and CDT, where the community reproductive number is far greater than the admission reproductive number, $R_{C}$ is estimated precisely, whereas median estimates for $R_{H}$ tend to be overestimated. The inflated estimates for the deme with the lower reproductive number can, to some extent, be explained by the prior distribution used: the signal enclosed within the transmission tree is less informative for the deme with a lower *R* and subsequently the estimates are being influenced by the prior distribution. As we used uniform prior distribution between 0 and 100, the inferred estimates tend to be elevated compared to the true value. Albeit for scenarios ET and CDT the median estimates for $R_{H}$ are overestimated, the overall tendency is inferred robustly and admission reproductive number estimates are evidently lower than community reproductive number estimates for each simulation replicate.

Principally, the proportion of the infected population being sampled in the community (i.e., 1.0%, 0.1% or 0.01%) seemed to have only a marginal impact on the transmission rate estimates, as long as bacterial genomes from the community were represented in the sample. For the majority of the scenarios no prominent differences were detected between the different sampling schemes. While the results appear rather robust regarding the proportion of samples taken from the community, it should be noted that this applies only when at least some infected individuals are being sampled outside the hospital environment. Only taking samples from the hospital results in heavily biased hospital transmission rates and hence samples from hospital should always be analyzed together with genomic data from the surrounding community. Additionally, it‘s important to emphasize that while our findings suggest that even the lowest community sampling proportion of 0.01% yielded relatively robust transmission rate estimates in many simulated scenarios, this should not be considered as a universally accepted threshold for community sampling in empirical studies. Instead, the robustness of transmission rate estimates derived from real-world data should ideally always be further validated through additional sensitivity analyses.

All substitution model parameters estimated as part of the Bayesian inference – namely, the transition/transversion ratio (*κ*), the shape parameter of the gamma distribution (*α*), and the nucleotide frequencies ($\pi_{A}$, $\pi_{C}$, $\pi_{G}$, and $\pi_{T}$) – were inferred with high accuracy across all simulation scenarios (S3–S5 Tables).

## Discussion

Whole-genome sequencing of bacterial strains in the hospital is important particularly for strain identification and outbreak investigation, and is becoming part of routine disease surveillance in many hospitals. The field of phylodynamics has developed rapidly over the last two decades, yielding methods that can shed light on the transmission dynamics of a range of pathogens from a variety of contexts. Nosocomial outbreaks are crucial to be contained quickly - or avoided altogether - as hospitalized patients are often immunocompromised and thus particularly vulnerable to infectious diseases. Outbreak control and containment are often more difficult once prevalence is high and infection is well established. Hence, it is central to ascertain to what extent infections observed in the hospital are caused by transmissions occurring in the hospital or in the community. In this study, we simulated synthetic bacterial sequences under a range of simulation scenarios to investigate the potential for phylodynamic analysis for nosocomial bacterial outbreaks. Assuming hospital admission and discharge rates as observed in the University Hospital Zurich in Switzerland, our results show that in relatively simple *Staphylococcus aureus*-like scenarios with very high within-hospital transmission, phylodynamic inference of the transmission dynamics within the hospital and the community performs well.

With low or moderate within-hospital transmission, however, it is difficult to disentangle the transmission dynamics in the hospital from those in the community. If the within-hospital transmission rate is as low or lower than the community transmission rate, estimation of the former results in a heavy bias. This bias, in this context, comes with large 95% HPD intervals indicating that the resulting low number of synthetic hospital sequences are not informative enough to estimate the hospital transmission rate with high precision. Although we would expect this bias to disappear if the proportion of sequenced hospital genomes was close to 100%, achieving this would be very costly and unrealistic in most real-world contexts. Furthermore, for scenarios with highly distinct admission and community reproductive numbers, a similar tendency can be observed and reproductive number estimates are higher than the true value particularly for the deme with lower reproductive numbers. It should be noted, however, that despite the absolute values being overestimated, the qualitative regime of high $R_{C}$ and low $R_{H}$ (i.e., self-sustained transmission in the community, but not in the hospital) is correctly inferred in each scenario. Nevertheless, researchers attempting equivalent analyses of real data need to carefully evaluate the posterior distributions of the estimated parameters and the corresponding 95% HPD intervals. Part of this evaluation can be performed by comparing prior and posterior distributions. However, while we can sample from a distribution independent of the sequence data, it will still be conditioned on the number of samples and their collection dates, which is a caveat of this method. When only point estimates are obtained, there is a danger of falsely concluding that within-hospital transmission drives an outbreak, as seen, for example, for the scenario where equal transmission rates are assumed. If only median estimates of transmission rates are being evaluated, dynamics appear to be heavily dominated by the within-hospital transmissions, whereas, in reality, posterior distributions for $\lambda_{H}$ and $\lambda_{C}$ are largely overlapping, which should be interpreted as absence of evidence for different dynamics between the two settings.

When limiting the sampling process to hospital cases only, for both hospital-driven and equal transmission scenarios the hospital transmission rate is notably underestimated. Moreover, for the community-driven scenario the median estimates for $\lambda_{H}$ are somewhat underestimated, if the sampling proportion within the hospital is known (scenario CDT’). If, however, $s_{H}$ remains unknown (i.e., scenario CDT”), the inferred hospital transmission rates reflect the transmission dynamics within the community, that is, $\lambda_{H}$ is overestimated. Fixing one of the within-compartment rates for each compartment prevents identifiability issues caused by potential within-compartment parameter correlations [48]. We also fixed the migration rates to their true values since they are very likely to be known in this context. With lower or higher community sampling and $s_{H}$ known, $\lambda_{H}$ estimates are close to the truth, but the HPD intervals become too narrow. Additionally, in scenario CDT” estimates for $s_{H}$ are closer to the true community sampling proportion than to the true hospital sampling proportion. Altogether, this suggests that the patterns of hospital dynamics are superseded by the strong signal of community transmission enclosed within the transmission tree. Accounting for the rate of patients entering the hospital in an unstructured phylodynamic model is not unambiguous with currently available approaches. A better approach for hospital-only phylodynamic analyses remains a challenge for future work. We employ a scheme that discards epidemics that do not reach a specified number of samples, which can introduce bias with reproduction numbers below the epidemic threshold of 1 as shown in simulation studies using one-deme birth–death–sampling models (e.g., *bdsky* in [50] and bdskyλ in [51]). Here, the same overall reproduction number of 1.2 was used, and the sampling threshold was applied to the sum of hospital and community samples, not to individual demes. Hence, as we always have a deme with a reproduction number well above 1, we do not expect such a bias here.

This work has several limitations. A key constraint is our focus on a single pathogen, *Staphylococcus aureus*, with simulations designed to reflect its specific transmission dynamics. While similar approaches could be extended to other clinically relevant bacteria, such as *Pseudomonas spp.*, this would require pathogen-specific simulation frameworks that account for differences in evolutionary and epidemiological properties. Moreover, in the case of *S. aureus*, we analysed synthetic bacterial genomes with a shared transmission history implying that within each simulation replicate we are tracing a single lineage spreading across hospital and community. A more plausible scenario would be, however, coexistence of different HA-MRSA and CA-MRSA associated lineages [52,53]. Furthermore, when applying phylodynamic approach for the analysis of real data, researchers need to identify transmission clusters first [54]. Cluster identification was not part of this study. Instead, we used the information on transmission chains from our simulations. It should also be emphasized that we consider the phylogeny to be a good representation of the transmission tree, for alternate approaches, see [55,56].

Another limitation of this study is that our approach does not incorporate the effects of homologuous recombination. Important steps towards the inclusion of recombination in phylodynamics have been and continue to be taken [57–59]. Furthermore, we only model “core genomes,” although the analysis of mobile genetic elements such as plasmids could give further insight into nosocomial transmission dynamics [60–63]. That is, we focus on relatively simple analyses assuming that any recombining sites and mobile genetic elements are removed prior to the phylodynamic analysis. Although we have not addressed microbial resistance here, our results likely hold for a range of drug sensitive and resistant pathogens. Future work shall make use of more complex evolutionary processes to increase the statistical power in phylodynamic inference and improve epidemiological surveillance of nosocomial pathogens, including drug-resistant microorganisms [64].

We use the exponential distribution, which can result in very short generation times, as opposed, e.g., to the gamma distribution. However, this is a common simplification shared by most compartmental models due to its memoryless property and to balance simplicity with practical modeling challenges [65]. Finally, the population dynamics in the hospital are simplified in our model as we neither account for population structure within the hospital (e.g., different hospital wards), nor the variability in the time to be discharged, which depends, e.g., on patient and disease characteristics and hospital type. Additionally, although healthcare workers (HCWs) and visitors can contribute to transmission between hospital and community, these pathways are not included in our current simulation framework. Due to strict infection prevention practices, such as effective hand hygiene, such transmission routes are generally minimized. However, hand hygiene compliance can vary considerably depending on, for instance, the role of the HCW and the clinical context [66–68], making it challenging to quantify the extent to which HCWs or visitors may facilitate bacterial spread between the hospital and the surrounding community. By not including a susceptible compartment an effectively infinite susceptible population is implicitly assumed, which is reasonable for community-level transmission dynamics, but not for the hospital setting where the number of susceptible individuals is limited by the total patient and staff population, which may affect transmission dynamics and potential outbreak size. While the method‘s performance under a high degree of heterogeneity in infection dynamics could theoretically be evaluated through additional simulations, even the most advanced simulation scenarios cannot fully replicate the complexity of real-world data. Therefore, the applicability of phylodynamic inference in investigating nosocomial bacterial outbreaks, particularly in the context of variability in population structure and length of hospital stays, as well as transmissions involving HCWs and visitors, requires further validation using empirical *S. aureus* genomic data combined with transmission histories obtained, e.g., through contact tracing. Such data would then also allow comparison with other methods that combine genomic data with transmission history [69,70]. Although numerous whole-genome sequences from hospitalized patients have been collected in observational studies [26,27], studies providing sufficient numbers of temporally corresponding genomes reflecting the local disease dynamics in the community are, to our knowledge, lacking. Since our findings suggest that genomic sequences from the surrounding community are crucial for reliable phylodynamic inference, further evaluation of the generalizability of our results is currently hindered by the scarcity of real-world data, making this an important avenue for future research.

Another promising research direction could involve using phylodynamics to assess the impact of external factors on disease transmission, similar to studies on SARS-CoV-2 [71,72]. This approach could potentially provide insights into how environmental factors, such as implementing strict control strategies in healthcare facilities after detecting initial bacterial infections, influence disease transmission within the surrounding community. This is particularly significant, as nosocomial transmissions are estimated to account for a considerable proportion of community infections in low-prevalence countries [73]. However, robust phylodynamic analyses with additional layers of complexity, e.g., to test the impact of interventions, would require larger empirical datasets than currently available. Clearly, more *S. aureus* whole-genomes are needed not only to validate the findings of this study but also to support future research addressing more complex evolutionary dynamics.

As the number of bacterial genomes increases in the coming years, our study provides several guidelines for their interpretation. Our results show that phylodynamic approaches based on whole genome sequencing can provide accurate estimates of the transmission dynamics of bacterial pathogens in hospitals and the surrounding community. A good understanding of the patient dynamics within the hospital as well as the transmission dynamics in the community are instrumental to phylodynamic analysis of bacterial outbreaks within the hospital, as many infections related to nosocomial outbreaks will not be observed within the hospital due to fast discharge rates. Thus, it is essential that bacterial genomes from the surrounding community are included in phylodynamic analyses, even if their primary goal is to quantify nosocomial transmission. This could be facilitated by a combination of entry screenings and the inclusion of genomic data from wastewater surveillance [74].

## Supporting information

S1 Fig Schematic illustration of true transmission tree vs. sampled transmission tree. (EPS)

S2 FigPosterior distributions for transmission rates $\lambda_{C}$ for simulation scenarios HDT (a)–(c) under community sampling proportion of $s_{C}=0.01$.(EPS)

S3 FigPosterior distributions for transmission rates $\lambda_{C}$ for simulation scenarios HDT (a)–(c) community sampling proportion of $s_{C}=0.0001$.(EPS)

S4 FigPosterior distributions for transmission rates $\lambda_{C}$ and $\lambda_{H}$ for simulation scenario ET under community sampling proportions of $s_{C}=0.01$ and $s_{C}=0.0001$.(EPS)

S5 FigPosterior distributions for transmission rates $\lambda_{C}$ and $\lambda_{H}$ for simulation scenario CDT under community sampling proportions of $s_{C}=0.01$ and $s_{C}=0.0001$.(EPS)

S6 FigPosterior distributions for community reproduction number ($R_{C}$) and admission reproduction number ($R_{H}$) for simulation scenarios HDT (a)–(c), ET, and CDT under community sampling proportion of $s_{C}=0.01$.(EPS)

S7 FigPosterior distributions for community reproduction number ($R_{C}$) and admission reproduction number ($R_{H}$) for simulation scenarios HDT (a)–(c), ET, and CDT under community sampling proportion of $s_{C}=0.001$.(EPS)

S8 FigPosterior distributions for community reproduction number ($R_{C}$) and admission reproduction number ($R_{H}$) for simulation scenarios HDT (a)–(c), ET, and CDT under community sampling proportion of $s_{C}=0.0001$.(EPS)

S1 Table Summary of the Bayesian posterior inference results for scenarios HDT (a) sensitivity 1 and 2. (PDF)

S2 TableSummary of posterior odds and transmission rate ratios between community ($\lambda_{C}$) and hospital ($\lambda_{H}$) settings across different transmission scenarios and community sampling rates ($s_{C}$).(PDF)

S3 TableSummary of Bayesian posterior inference results for substitution model parameters from each simulation scenario, assuming a community sampling rate of $s_{C}$ = 0.01.(PDF)

S4 TableSummary of Bayesian posterior inference results for substitution model parameters from each simulation scenario, assuming a community sampling rate of $s_{C}$ = 0.001.(PDF)

S5 TableSummary of Bayesian posterior inference results for substitution model parameters from each simulation scenario, assuming a community sampling rate of $s_{C}$ = 0.0001.(PDF)

S1 Text Computing community transmission numbers. (PDF)

S2 TextImproving convergence for simulation scenario HDT (a) ($\lambda_{H}$ = 36.0$y^{-1}$).(PDF)

S3 TextSimulation replicates with distinctive inferred δ estimates (replicates 3136 & 3189 and 3112 & 3152, for scenarios $\lambda_{H}$ = 45.0$y^{-1}$ and $\lambda_{H}$ = 49.5$y^{-1}$, respectively).(PDF)
