# Supplementary material for: Fast hospital discharge rates blur within-hospital ‘transmission footprint’ in bacterial genomes, as showcased with Staphylococcus aureus
Source: PLoS Comput Biol. 2026 Mar 16;22(3):e1013982. doi: 10.1371/journal.pcbi.1013982 (PMC13008258; doi:10.1371/journal.pcbi.1013982)
Supplement: S1 Table — (PDF) [file pcbi.1013982.s009.pdf]

Fast hospital discharge rates blur within-hospital 'transmission footprint' in bacterial genomes, as showcased with *Staphylococcus aureus*

Supplementary table S1. Summary of the Bayesian posterior inference results for scenarios HDT (a) sensitivity 1 and 2 with $s_H = 0.1$ and $s_H = 0.05$, respectively. For comparison, results from the main HDT (a) analysis by assuming $s_H = 0.2$, are also shown. The column labelled 'ESS > 200' indicates the number of replicates (out of 100) in which all parameters had an effective sample size (ESS) of at least 200. Epidemiological parameters not listed in the third column (e.g., admission and discharge rates) were fixed to their true values. The 'Relative error' column reflects the average relative absolute deviation, calculated as |median−truth|/truth. The 'Relative bias' column shows the average relative deviation of the median estimate from the true value, calculated as (median−truth)/truth. Relative 95% highest posterior density (HPD) widths are computed as (upper bound−lower bound)/truth. The '95% HPD accuracy' column indicates the number of replicates in which the 95% HPD interval included the true value for each parameter.

Scenario	ESS > 200	Parameter	Truth	Median	Relative error	Relative bias	Relative HPD width	95% HPD accuracy
HDT (a)<br>$s_C = 0.01$<br>$s_H = 0.2$	93	λ_C<br>λ_H<br>δ	1.00<br>36.00<br>1.00	0.99<br>35.79<br>0.98	0.12<br>0.03<br>0.11	-0.01<br>-0.01<br>-0.02	0.71<br>0.15<br>0.66	97<br>98<br>98
HDT (a)<br>sensitivity 1<br>$s_C = 0.01$<br>$s_H = 0.1$	98	λ_C<br>λ_H<br>δ	1.00<br>36.00<br>1.00	0.98<br>35.90<br>0.98	0.15<br>0.04<br>0.13	-0.02<br>0.00<br>-0.02	0.73<br>0.15<br>0.67	96<br>95<br>96
HDT (a)<br>sensitivity 2<br>$s_C = 0.01$<br>$s_H = 0.05$	96	λ_C<br>λ_H<br>δ	1.00<br>36.00<br>1.00	0.98<br>35.86<br>0.98	0.14<br>0.03<br>0.13	-0.02<br>0.00<br>-0.02	0.72<br>0.15<br>0.66	95<br>95<br>94
HDT (a)<br>$s_C = 0.001$<br>$s_H = 0.2$	91	λ_C<br>λ_H<br>δ	1.00<br>36.00<br>1.00	0.96<br>35.98<br>0.96	0.21<br>0.02<br>0.19	-0.04<br>0.00<br>-0.05	0.94<br>0.10<br>0.85	93<br>99<br>90
HDT (a)<br>sensitivity 1<br>$s_C = 0.001$<br>$s_H = 0.1$	82	λ_C<br>λ_H<br>δ	1.00<br>36.00<br>1.00	0.98<br>35.92<br>0.98	0.19<br>0.02<br>0.17	-0.02<br>0.00<br>-0.02	0.97<br>0.10<br>0.89	94<br>93<br>95
HDT (a)<br>$s_C = 0.001$<br>sensitivity 2<br>$s_H = 0.05$	82	λ_C<br>λ_H<br>δ	1.00<br>36.00<br>1.00	0.95<br>36.01<br>0.94	0.23<br>0.02<br>0.22	-0.05<br>0.00<br>-0.06	0.97<br>0.10<br>0.87	88<br>93<br>84
HDT (a)<br>$s_C = 0.0001$<br>$s_H = 0.2$	86	λ_C<br>λ_H<br>δ	1.00<br>36.00<br>1.00	0.98<br>35.90<br>0.97	0.21<br>0.02<br>0.20	-0.02<br>0.00<br>-0.03	1.07<br>0.10<br>0.96	95<br>98<br>92
HDT (a)<br>$s_C = 0.0001$<br>sensitivity 1<br>$s_H = 0.1$	79	λ_C<br>λ_H<br>δ	1.00<br>36.00<br>1.00	0.91<br>35.91<br>0.9	0.24<br>0.02<br>0.23	-0.09<br>0.00<br>-0.10	1.04<br>0.09<br>0.93	92<br>95<br>92
HDT (a)<br>sensitivity 2<br>$s_C = 0.0001$<br>$s_H = 0.05$	68	λ_C<br>λ_H<br>δ	1.00<br>36.00<br>1.00	0.94<br>35.97<br>0.93	0.26<br>0.02<br>0.25	-0.06<br>0.00<br>-0.07	1.08<br>0.09<br>0.97	96<br>99<br>96
