# Supplementary material for: Fast hospital discharge rates blur within-hospital ‘transmission footprint’ in bacterial genomes, as showcased with Staphylococcus aureus
Source: PLoS Comput Biol. 2026 Mar 16;22(3):e1013982. doi: 10.1371/journal.pcbi.1013982 (PMC13008258; doi:10.1371/journal.pcbi.1013982)
Supplement: S2 Table — (PDF) [file pcbi.1013982.s010.pdf]

Fast hospital discharge rates blur within-hospital 'transmission footprint' in bacterial genomes, as showcased with *Staphylococcus aureus*

Supplementary table S2. Summary of posterior odds and transmission rate ratios between community (λ_C) and hospital (λ_H) settings across different transmission scenarios and community sampling rates (s_C). The table reports the median and interquartile ranges (IQR) of posterior OR, median and IQR of λ_C/λ_H, and the proportion of replicates with posterior odds >1. For each scenario, estimates were calculated across simulation replicates yielding ESS>200 for all parameters included in the model.

Community sampling rate	Scenario	Median posterior odds ratio	IQR posterior odds ratio	Median λ_C/λ_H	IQR λ_C/λ_H	Proportion of posterior odds >1.0
$s_C = 0.01$	HDT (a)	1000	1000–3332.67	0.03	0.03–0.03	1
	HDT (b)	1000	1000–1000	0.02	0.02–0.02	1
	HDT (c)	1000	1000–1000	0.02	0.02–0.03	1
	ET	12.81	9.59–21.14	0.55	0.34–0.83	1
	CDT	13.4	9.37–22.83	0.55	0.38–0.87	1
$s_C = 0.001$	HDT (a)	1000	1000–10000	0.03	0.02–0.03	1
	HDT (b)	1000	1000–1000	0.02	0.02–0.03	1
	HDT (c)	1000	1000–1000	0.03	0.02–0.04	1
	ET	8.83	5.55–13.37	0.92	0.55–1.72	1
	CDT	8.14	5.29–14.7	0.97	0.64–1.58	1
$s_C = 0.0001$	HDT (a)	1000	1000–4999.5	0.03	0.02–0.03	1
	HDT (b)	1000	1000–1000	0.03	0.02–0.03	0.99
	HDT (c)	1000	1000–1000	0.04	0.03–0.05	0.99
	ET	9.43	6.23–16.3	0.91	0.48–1.41	1
	CDT	9.37	6.19–14.52	0.82	0.53–1.14	1
