# Supplementary material for: Fast hospital discharge rates blur within-hospital ‘transmission footprint’ in bacterial genomes, as showcased with Staphylococcus aureus
Source: PLoS Comput Biol. 2026 Mar 16;22(3):e1013982. doi: 10.1371/journal.pcbi.1013982 (PMC13008258; doi:10.1371/journal.pcbi.1013982)
Supplement: S4 Table — (PDF) [file pcbi.1013982.s012.pdf]

Fast hospital discharge rates blur within-hospital 'transmission footprint' in bacterial genomes, as showcased with *Staphylococcus aureus*

Supplementary table S4. Summary of Bayesian posterior inference results for substitution model parameters from each simulation scenario, assuming a community sampling rate of $s_C = 0.001$. Scenario abbreviations are as follows: HDT = hospital-driven transmission, ET = equal transmission, and CDT = community-driven transmission. κ represents the transition/transversion ratio in the HKY substitution model, while α is the shape parameter of gamma distribution describing among-site rate heterogeneity. π_A, π_C, π_G and π_T denote the nucleotide base frequencies. The 'Relative bias' column shows the average relative deviation of the median estimate from the true value, calculated as (median−truth)/truth. The 'Relative error' column reflects the average relative absolute deviation, calculated as |median−truth|/truth. Relative 95% highest posterior density (HPD) widths are computed as (upper bound−lower bound)/truth. The '95% HPD accuracy' column indicates the number of replicates in which the 95% HPD interval included the true value for each parameter.

Scenario	Parameter	Truth	Median	Relative error	Relative bias	Relative HPD width	95% HPD accuracy
HDT (a)	κ	4.04	4.07	0.04	0.01	0.17	93
	α	1.00	1.06	0.19	0.06	3.4	100
	π_A	0.34	0.34	0.0	0.0	0.0	93
	π_C	0.16	0.16	0.0	0.0	0.01	98
	π_G	0.16	0.16	0.0	0.0	0.01	92
	π_T	0.34	0.34	0.0	0.0	0.0	91
HDT (b)	κ	4.04	4.09	0.1	0.01	0.53	94
	α	1.00	0.72	0.28	-0.28	3.02	100
	π_A	0.34	0.34	0.0	0.0	0.0	97
	π_C	0.16	0.16	0.0	0.0	0.01	93
	π_G	0.16	0.16	0.0	0.0	0.01	97
	π_T	0.34	0.34	0.0	0.0	0.0	93
HDT (c)	κ	4.04	4.13	0.14	0.02	0.78	94
	α	1.00	0.7	0.3	-0.3	3.02	100
	π_A	0.34	0.34	0.0	0.0	0.0	96
	π_C	0.16	0.16	0.0	0.0	0.01	97
	π_G	0.16	0.16	0.0	0.0	0.01	96
	π_T	0.34	0.34	0.0	0.0	0.0	94
ET	κ	4.04	4.02	0.02	0.0	0.11	97
	α	1.00	1.42	0.46	0.42	3.59	100
	π_A	0.34	0.34	0.0	0.0	0.0	95
	π_C	0.16	0.16	0.0	0.0	0.01	96
	π_G	0.16	0.16	0.0	0.0	0.01	97
	π_T	0.34	0.34	0.0	0.0	0.0	92
CDT	κ	4.04	4.03	0.02	0.0	0.11	97
	α	1.00	1.41	0.45	0.41	3.61	100
	π_A	0.34	0.34	0.0	0.0	0.0	95
	π_C	0.16	0.16	0.0	0.0	0.01	95
	π_G	0.16	0.16	0.0	0.0	0.01	96
	π_T	0.34	0.34	0.0	0.0	0.0	94
