# Supplementary material for: Fast hospital discharge rates blur within-hospital ‘transmission footprint’ in bacterial genomes, as showcased with Staphylococcus aureus
Source: PLoS Comput Biol. 2026 Mar 16;22(3):e1013982. doi: 10.1371/journal.pcbi.1013982 (PMC13008258; doi:10.1371/journal.pcbi.1013982)
Supplement: S1 Text — (PDF) [file pcbi.1013982.s014.pdf]

Fast hospital discharge rates blur within-hospital 'transmission footprint' in bacterial genomes, as showcased with *Staphylococcus aureus*

Supplementary text S1. Computing community transmission numbers. The within-community transmission rates $\lambda_C = 1.0y^{-1}$, $\lambda_C = 0.69y^{-1}$, and $\lambda_C = 0.07y^{-1}$ were computed with next generation matrix [1] as follows. Calculations were performed using SageMath [2].

Next-generation matrix FV^{-1}

$$F = \begin{bmatrix} \lambda_C & 0 & 0 \\ 0 & \lambda_H & 0 \\ 0 & 0 & 0 \end{bmatrix} \quad \text{and} \quad V = \begin{bmatrix} \delta_C + m_{CH} & -m_{CH} & 0 \\ -m_{HC} & \delta_H + m_{HC} & 0 \\ 0 & 0 & -\delta_C - \delta_H \end{bmatrix}$$

$$V^{-1} = \begin{bmatrix} \frac{\delta_H + m_{CH}}{\delta_C(\delta_H + m_{CH}) + \delta_H m_{HC}} & \frac{m_{HC}}{\delta_C(\delta_H + m_{CH}) + \delta_H m_{HC}} & 0 \\ \frac{m_{CH}}{\delta_C(\delta_H + m_{CH}) + \delta_H m_{HC}} & \frac{\delta_C + m_{HC}}{\delta_C(\delta_H + m_{CH}) + \delta_H m_{HC}} & 0 \\ 0 & 0 & -\frac{1}{\delta_C + \delta_H} \end{bmatrix}$$

$$FV^{-1} = \begin{bmatrix} \frac{\lambda_C \delta_H + \lambda_C m_{HC}}{\delta_C \delta_H + \delta_H m_{CH} + \delta_C m_{HC}} & \frac{\lambda_C m_{CH}}{\delta_C \delta_H + \delta_H m_{CH} + \delta_C m_{HC}} \\ \frac{\lambda_H m_{HC}}{\delta_C \delta_H + \delta_H m_{CH} + \delta_C m_{HC}} & \frac{\lambda_H \delta_C + \lambda_H m_{CH}}{\delta_C \delta_H + \delta_H m_{CH} + \delta_C m_{HC}} \end{bmatrix}$$

Assuming an overall reproduction number of $R_0 = 1.2$, we can then compute λ_C for $\lambda_H = 36.0y^{-1}$, $\lambda_H = 45.0y^{-1}$, and $\lambda_H = 49.5y^{-1}$, respectively, with:

$$R_0 = \max\left(eigenvalue(FV^{-1})\right) \coloneqq 1.2.$$

Due to the discharge rate being much higher than the transmission rates in any realistic community-driven or equal transmission scenario, the highest eigenvalue of the next-generation matrix is driven by the community transmission rate: With $\lambda_C = 1.2y^{-1}$ any $\lambda_H <= 1.2y^{-1}$ results in an overall reproduction number of $R_0 \approx 1.2$. Hence, the hospital transmission rates λ_H were chosen to be equal ($\lambda_H = \lambda_C = 1.2y^{-1}$) in scenario ET and lower ($\lambda_H = 0.75y^{-1}$) in scenario CDT.

References

[1] Diekmann O, Heesterbeek JAP, Metz JA. On the definition and the computation of the basic reproduction ratio R0 in models for infectious diseases in heterogeneous populations. *Journal of mathematical biology*. 1990; 28(4):365–382.

[2] Szabó P, Galanda J. Sage math for education and research. In: 2017 15th International Conference on Emerging eLearning Technologies and Applications (ICETA); 2017. p. 1–4.
