# Supplementary material for: Fast hospital discharge rates blur within-hospital ‘transmission footprint’ in bacterial genomes, as showcased with Staphylococcus aureus
Source: PLoS Comput Biol. 2026 Mar 16;22(3):e1013982. doi: 10.1371/journal.pcbi.1013982 (PMC13008258; doi:10.1371/journal.pcbi.1013982)
Supplement: S2 Text — (PDF) [file pcbi.1013982.s015.pdf]

Fast hospital discharge rates blur within-hospital 'transmission footprint' in bacterial genomes, as showcased with *Staphylococcus aureus*

Supplementary text S2. Improving convergence for simulation scenario HDT (a) ($\lambda_H = 36.0y^{-1}$). For all simulation scenarios we assessed sufficient mixing for the Markov Chain Monte Carlo (MCMC) analysis by ensuring that effective samples size (ESS) values for all parameters included in the model reached the desired threshold of 200. Whereas for the majority of the simulation scenarios MCMC chain length of 10 million steps was sufficient to reach ESS > 200 for all parameters, this condition was not met for numerous replicates of simulation scenario HDT (a) with $\lambda_H = 36.0y^{-1}$. ESS values lower than 200 were obtained particularly for the following parameters: tree height, recovery rate, and the tree prior. For those replicates, for which any of the above-mentioned parameters yielded ESS values of 100 < ESS < 200, we increased the operator weight for the tree scaler to 10.0 and ran the MCMC chain for 20 million steps. This notably improved the convergence for the majority of replicates.

In addition, under all sampling schemes ($s_C = 0.01$, $s_C = 0.001$ and $s_C = 0.0001$), for scenarios HDT (a), HDT (a) sensitivity 1, and HDT (a) sensitivity 2 we detected replicates for which ESS values for the tree height were lower than 100. A closer examination of the simulated trees revealed that within the trees there are several consecutive transmission events within a relatively short time period directly or shortly after the pathogen has been introduced to the hospital for the first time (Figure A). Regarding the inference, the sequence of rapidly occurring transmission events makes it challenging to infer the correct origin of the outbreak which is subsequently reflected as a poor convergence for the tree height. As changing the operator weight and increasing the MCMC chain length, as described above, did not result in notable improvement in mixing, for the replicates given below, we further modified the scaling operator for the tree height such that optimisation was turned off `(optimise=false)`, and changed the scaling factor from 0.9 to 0.75, and we increased the chain length to 30 million steps.

List of simulation replicates for which ESS estimates less than 100 were obtained for the `TreeHeight` parameter:

HDT (a), i.e. assuming $s_H = 0.2$

$s_C = 0.01$: 3100, 3125*, 3146*, 3149*, 3161, 3168, 3173*, 3179*, 3183*, 3192, 3193.
$s_C = 0.001$: 3110, 3117, 3125*, 3134, 3139*, 3145*, 3146, 3147, 3156, 3162*, 3172, 3174*, 3186*, 3189*, 3192*, 3193, 3196*.
$s_C = 0.0001$: 3100, 3104, 3125*, 3134, 3145, 3147, 3149, 3150*, 3156, 3179, 3181*, 3185*, 3189.

HDT (a) sensitivity 1, i.e. assuming $s_H = 0.1$

$s_C = 0.01$: 3163
$s_C = 0.001$: 3117, 3125, 3134, 3145, 3146, 3147, 3149, 3156, 3164, 3168, 3171, 3179, 3193, 3196
$s_C = 0.0001$: 3125, 3134, 3145, 3146, 3149*, 3156, 3163, 3168, 3172*, 3179, 3192

HDT (a) sensitivity 2, i.e. assuming $\mathbf{s_H = 0.05}$

$s_C = 0.01$: 3163, 3168, 3170
$s_C = 0.001$: 3113, 3117, 3125, 3133, 3145, 3147*, 3149*, 3156, 3192*
$s_C = 0.0001$: 3104, 3121*, 3125, 3129, 3134, 3145, 3146, 3147, 3149*, 3156, 3163, 3170, 3171, 3172, 3179, 3186, 3187, 3196

For the simulation replicates indicated with an asterisk the above-mentioned modifications improved the convergence and ESS values above 200 were obtained for all the parameters of interest.

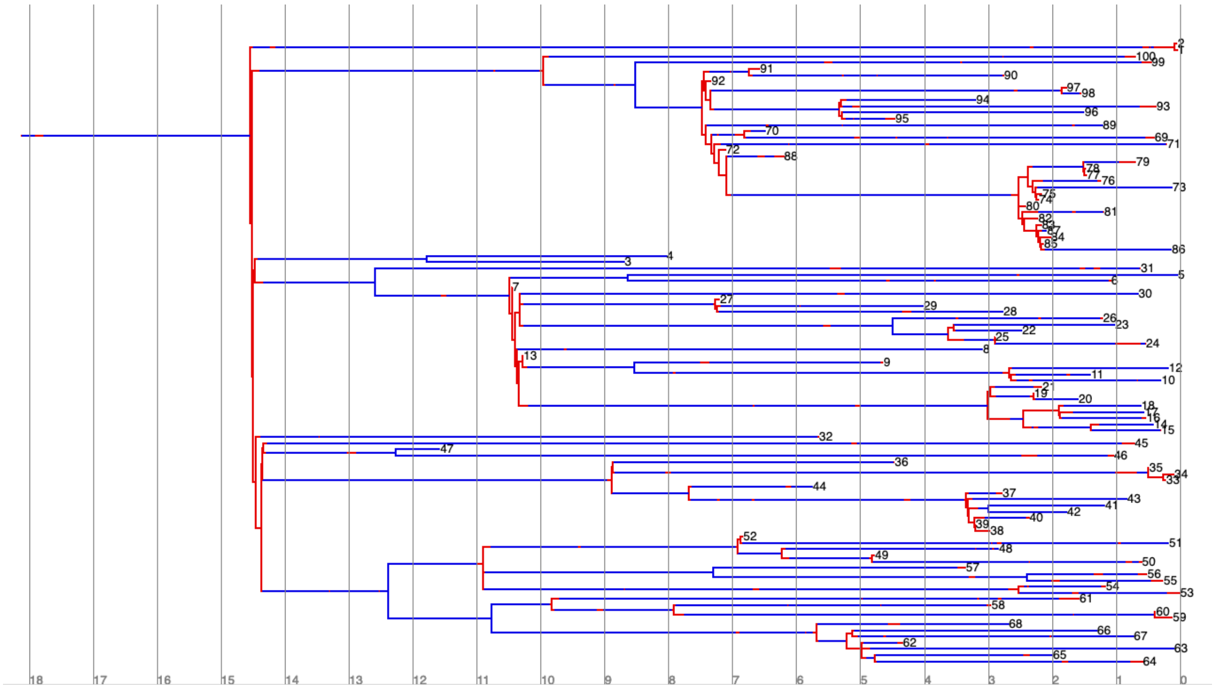

Figure A. Example of the simulated sampled tree with notably low ESS value for tree height parameter (simulation scenario $\lambda_H = 36.0y^{-1}$ with $s_C = 0.001$, simulation replicate 3110). Branches of the tree are colored according to the demes: blue color indicates community whereas red color denotes hospital. The x-axis represents time, here with the unit being years. Figure created with IcyTree [1].

To further confirm that modifying the tree scaling operator does not bias our results, we repeated the approach outlined in point a) for five replicates for which the ESS values for the tree height were above 200 after initial run. As visualized in the Figure B, operator changes do not have a substantial impact on the tree space sampled. Instead, the tree space plots (created with RWTY [2]) are comparable between the chains with and without the operator modification.

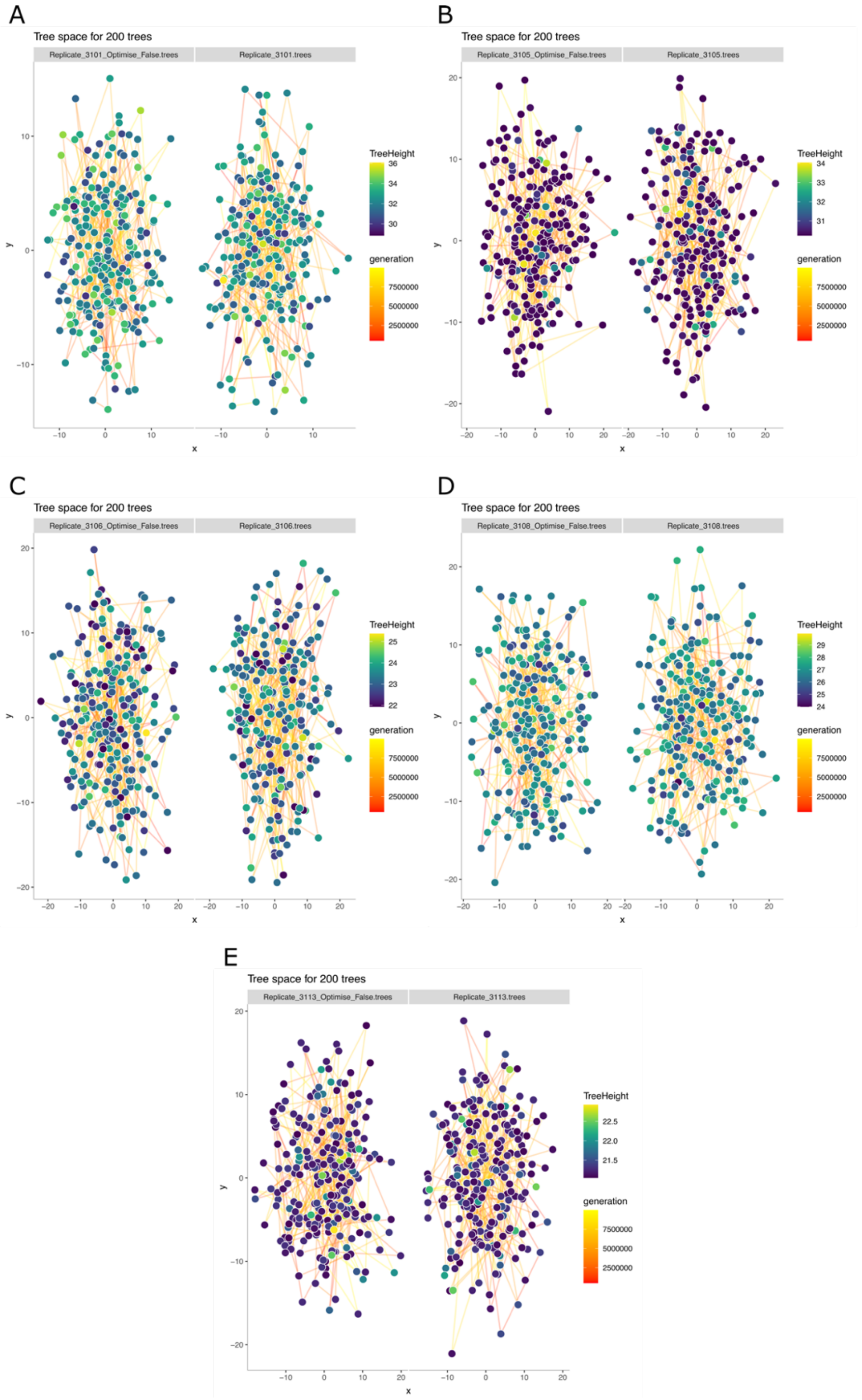

Figure B. Impact of the tree scaling operator changes on the tree space sampled. Plots represent the movement of the MCMC chain along the two-dimensional tree space for 200 trees. In each figure A–E, on the left the tree scaling operator is set to `optimise=false` whereas on the right the default of `optimise=true` is being used. Samples are colored according to the tree height parameter estimates. Results are represented for simulation replicates 3101, 3105, 3106, 3108 and 3113, all for which the ESS values for the tree height were above 200 after the initial run. Figure created with R package RWTY [2].

References

[1] Vaughan TG. IcyTree: rapid browser-based visualization for phylogenetic trees and networks. *Bioinformatics*. 2017; 33(15):2392–2394.

[2] Warren DL, Geneva AJ, Lanfear R. RWTY (R We There Yet): An R package for examining convergence of Bayesian phylogenetic analyses. *Molecular Biology and Evolution*. 2017; 34(4):1016–1020.
