# Supplementary material for: Fast hospital discharge rates blur within-hospital ‘transmission footprint’ in bacterial genomes, as showcased with Staphylococcus aureus
Source: PLoS Comput Biol. 2026 Mar 16;22(3):e1013982. doi: 10.1371/journal.pcbi.1013982 (PMC13008258; doi:10.1371/journal.pcbi.1013982)
Supplement: S3 Text — (PDF) [file pcbi.1013982.s016.pdf]

**Fast hospital discharge rates blur within-hospital 'transmission footprint' in bacterial genomes, as showcased with *Staphylococcus aureus***

**Supplementary text S3.** Simulation replicates with distinctive inferred $\delta$ estimates (replicates 3136 & 3189 and 3112 & 3152, for scenarios $\lambda_H = 45.0y^{-1}$ and $\lambda_H = 49.5y^{-1}$, respectively).

When assuming $s_C = 0.0001$ for both scenarios HDT (b) and HDT (c) for two simulation replicates we inferred notably higher posterior estimates for the rate to become non-infectious than the true value ($\delta = 1.0\, y^{-1}$) (Figure A). Whereas for all other replicates the posterior distributions for $\delta$ are narrow and centered around the true simulated value, for HDT (b) replicates 3136 and 3189 and for HDT (c) replicates 3112 and 3152 the distributions are wide with median estimates ranging between 31 and 66 (Figure A, Tables A and B). Correspondingly, for these four replicates the posterior distributions for transmission rates are wide and particularly for $\lambda_C$ inferred values are highly overestimated (Figures B and C, Tables A and B).

A closer examination of the simulated transmission trees revealed that for the replicates that yielded unexpectedly high $\delta$ estimates the onset of the outbreak occurs only after a rather long 'community' period without any observable transmissions (Figures D and E). We hypothesize that as a consequence of the notable delay between outbreak origins and first branching event in the tree it becomes challenging to infer the true transmission rate. Within the Bayesian inference framework, the resulting posterior distribution is largely reflecting the prior distribution. For all above mentioned parameters under scrutiny ($\delta$, $\lambda_H$ and $\lambda_C$) the prior distribution used was uniform (0, 100) and as shown in the Figures A–C, the inferred values for these parameters are widely distributed around the mean value of the prior distribution. To further test this, we performed additional analysis for simulation replicates 3136, 3189, 3112 and 3152 under the inference where the rate to become non-infectious was fixed to its' true value ($\delta = 1.0\, y^{-1}$). When fixing the $\delta = 1.0$, the posterior distributions for the transmission rates $\lambda_C$ and $\lambda_H$ overlap with true simulated value for all replicates 3136, 3189, 3112 and 3152 (Figures B and C, Tables A and B).

Despite for replicates 3136, 3189, 3112 and 3152 the MCMC chain converged and effective sample size (ESS) values above 200 were obtained for all parameters included in the analysis, these replicates were omitted from subsequent analysis.

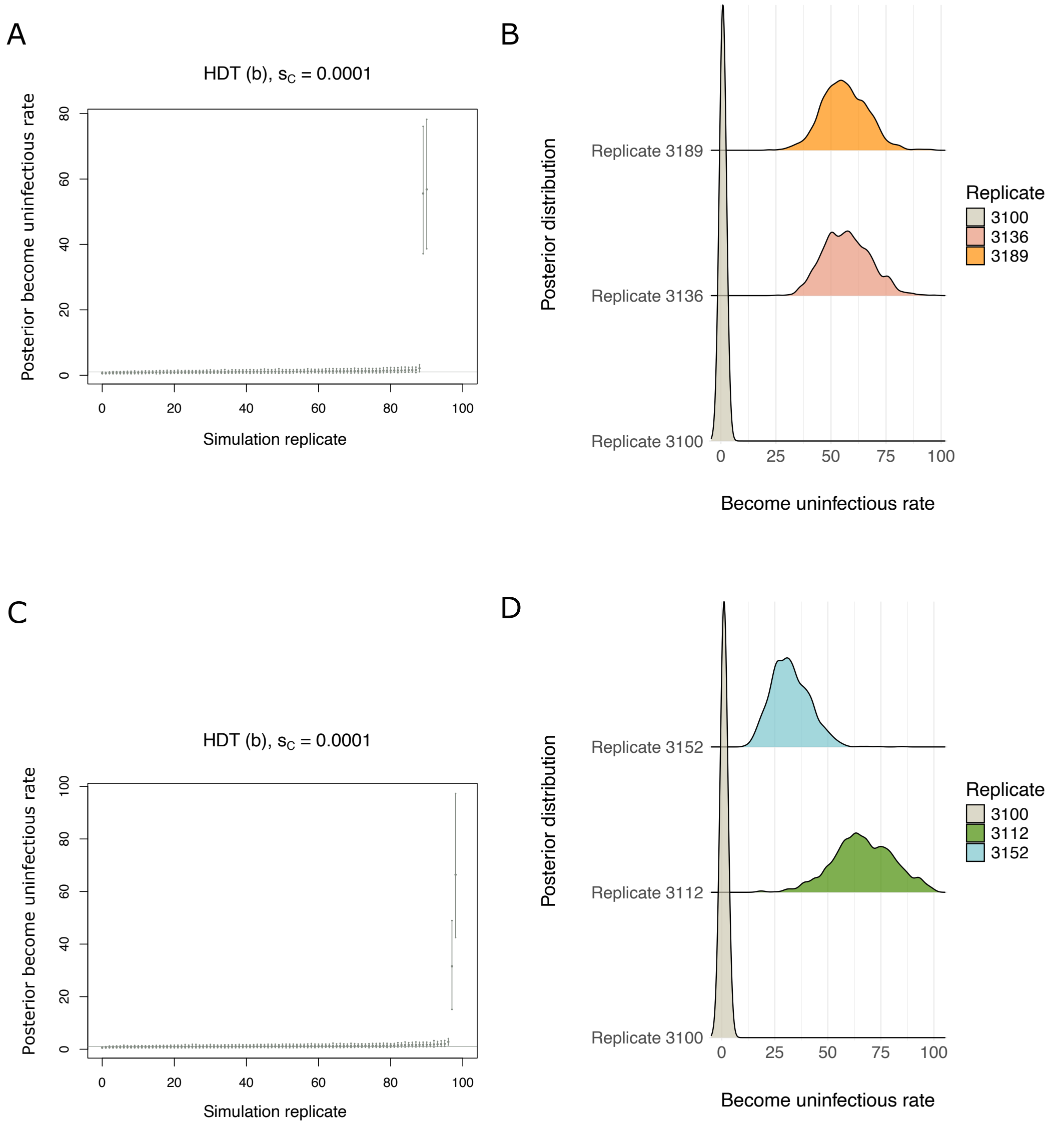


**Figure A.** Posterior distributions of $\delta$ for replicates 3136 and 3189 (upper panel) and replicates 3112 and 3152 (lower panel). In panels A and C each bar represents 95% highest posterior density interval from a simulation replicate with a point denoting the median estimate. Horizontal line indicates the true rate to become non-infectious value ($\delta = 1.0\,y^{-1}$). For replicates 3136 and 3189 as well as for replicates 3112 and 3152 estimated $\delta$ values are substantially higher than the true value. For these four replicates the posterior distributions are widely distributed (panels B and D) whereas for all the other simulation replicates posterior distributions for $\delta$ are tightly centered around value of 1.0 (simulation replicate 3100 shown as an example).

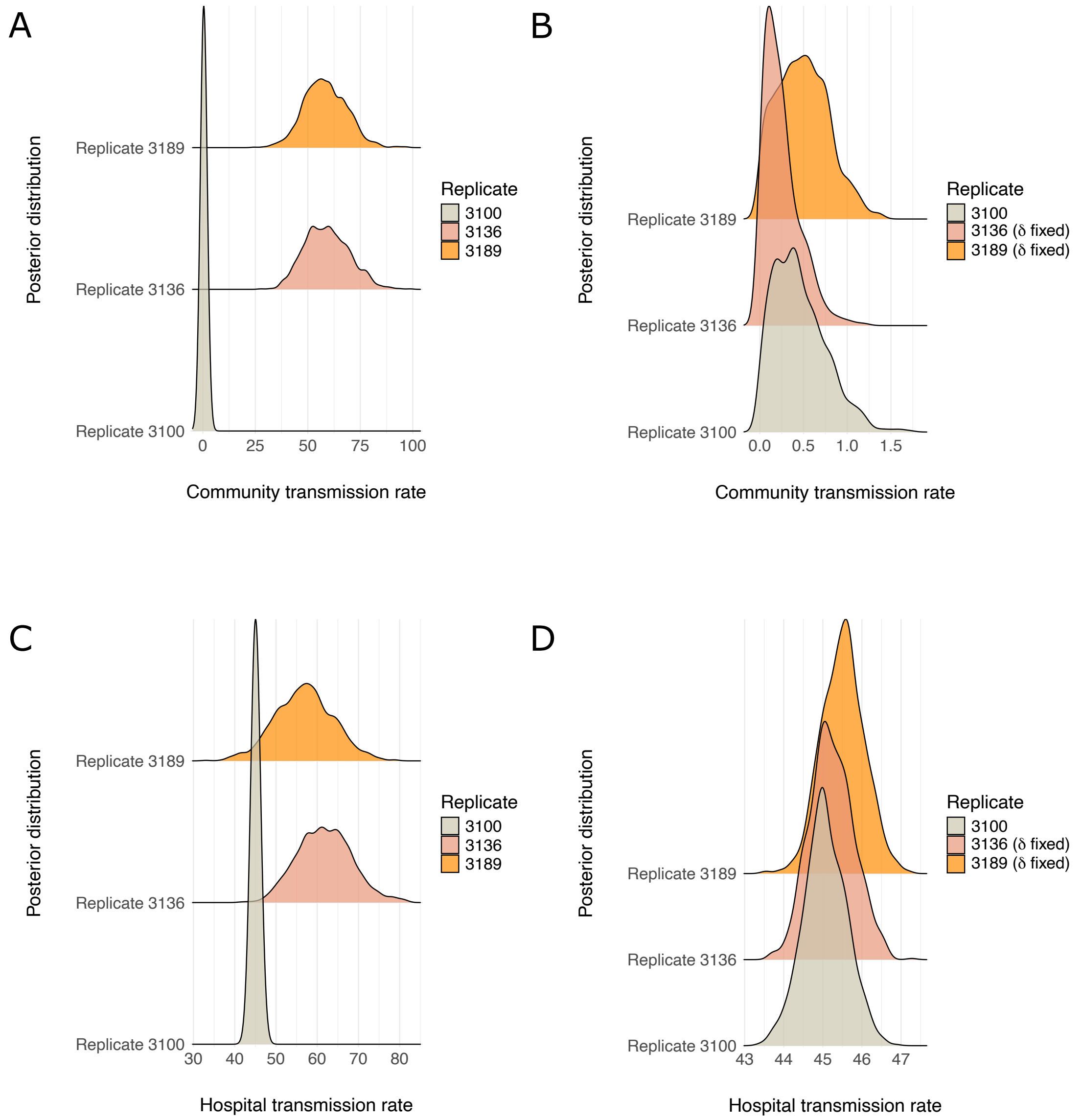


**Figure B.** Posterior distributions for $\lambda_C$ (upper panel) and $\lambda_H$ (lower panel) for simulation replicates 3136 and 3189 ($\lambda_H = 45.0y^{-1}$ and $s_C = 0.0001$). Panels A and C represent posterior distributions for $\lambda_C$ and $\lambda_H$, respectively. For comparison, posterior distributions for replicate 3100 are shown. Panels B and D represent posterior distributions for $\lambda_C$ and $\lambda_H$, respectively, under the inference where rate to become non-infectious is fixed to its' true value ($\delta = 1.0\ y^{-1}$).

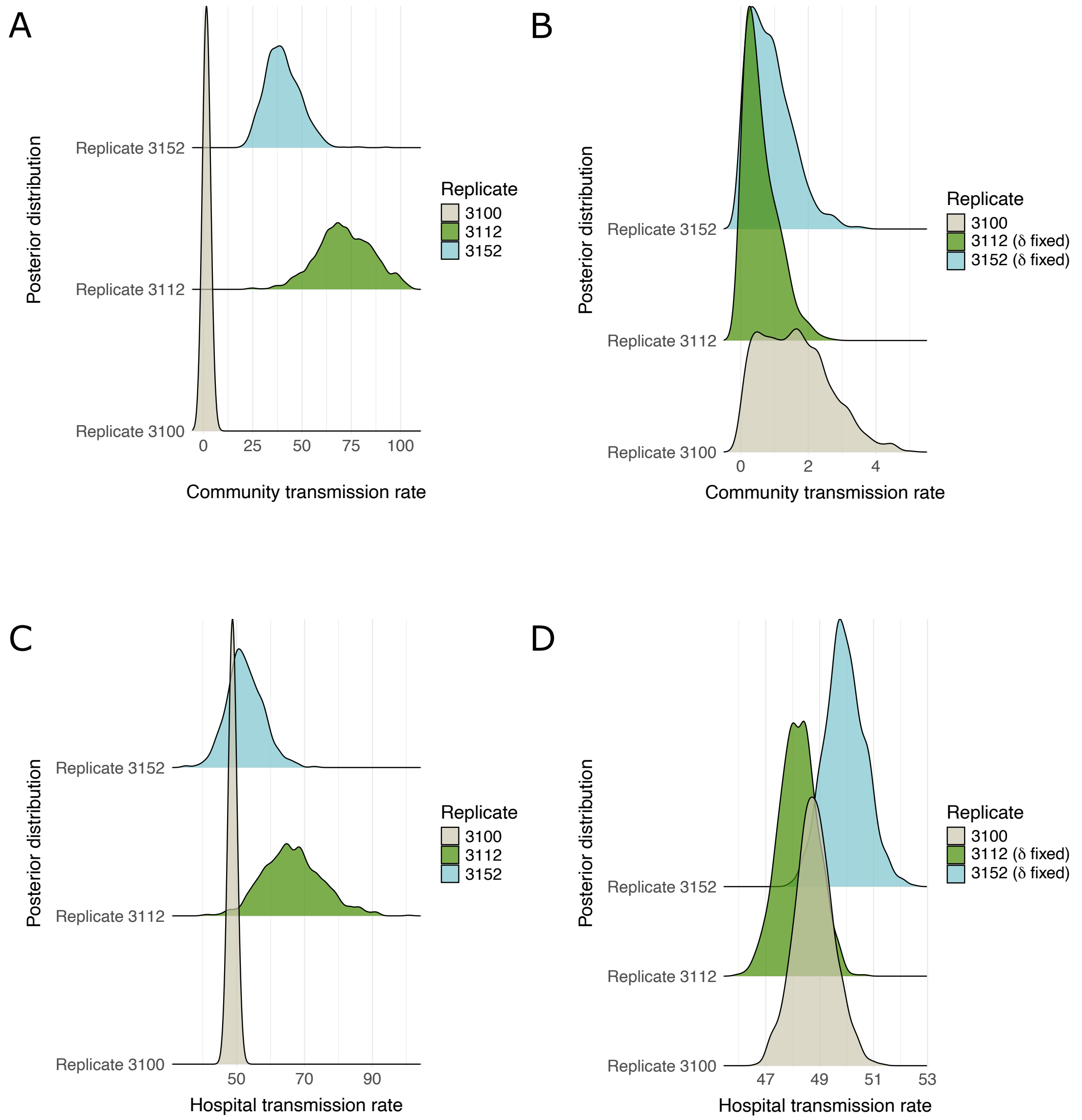


**Figure C**. Posterior distributions for $\lambda_C$ (upper panel) and $\lambda_H$ (lower panel) for simulation replicates 3112 and 3152 ($\lambda_H = 49.5y^{-1}$ and $s_C = 0.0001$). Panels A and C represent posterior distributions for $\lambda_C$ and $\lambda_H$, respectively. For comparison, posterior distributions for replicate 3100 are shown. Panles B and D represent posterior distributions for $\lambda_C$ and $\lambda_H$, respectively, under the inference where rate to become non-infectious is fixed to its' true value ($\delta = 1.0\ y^{-1}$).

**Table A.** Inferred median estimates for parameters $\delta$, $\lambda_H$ and $\lambda_C$ for replicates 3136 and 3189 ($\lambda_H = 45.0y^{-1}$ and $s_C = 0.0001$). For comparison, estimates for replicate 3100 are given. For individual replicates, the 95% highest posterior density (HPD) intervals are given in the parenthesis. When fixing the $\delta = 1.0$ within the Bayesian inference, the inferred posterior distributions for replicates 3136 and 3189 for the transmission rates $\lambda_C$ and $\lambda_H$ overlap with true simulated value. * Replicates 3136 and 3189 excluded.

| Parameter | Truth | Prior | Replicate 3136 | Replicate 3189 | Replicate 3100 | Replicates 3100–3199* |
|---|---|---|---|---|---|---|
| $\delta$ | 1.0 | Unif(0, 100) | 56.83 [38.67, 77.98] | 55.59 [37.16, 76.08] | 0.88 [0.55, 1.29] | 1.11 |
| $\lambda_C$ | 0.69 | Unif(0, 100) | 58.69 [40.53, 79.98] | 57.73 [39.42, 78.04] | 0.41 [$2x10^{-4}$, 1.02] | 1.00 |
| $\lambda_H$ | 45.0 | Unif(0, 100) | 61.68 [48.93, 74.31] | 56.40 [44.32, 72.08] | 45.04 [43.95, 46.12] | 44.97 |
| | | | | | | |
| $\delta$ | 1.0 | Fixed | 1.0 | 1.0 | – | – |
| $\lambda_C$ | 0.69 | Unif(0, 100) | 0.20 [$2x10^{-3}$, 0.64] | 0.48 [$5x10^{-3}$, 1.01] | – | – |
| $\lambda_H$ | 45.0 | Unif(0, 100) | 45.19 [44.14, 46.29] | 45.52 [44.57, 46.63] | – | – |

**Table B.** Inferred median estimates for parameters $\delta$, $\lambda_H$ and $\lambda_C$ for replicates 3112 and 3152 ($\lambda_H = 49.5y^{-1}$ and $s_C = 0.0001$). For comparison, estimates for replicate 3100 are given. For individual replicates, the 95% highest posterior density (HPD) intervals are given in the parenthesis. When fixing the $\delta = 1.0$ within the Bayesian inference, the inferred posterior distributions for replicates 3112 and 3152 for the transmission rates $\lambda_C$ and $\lambda_H$ overlap with true simulated value. * Replicates 3112 and 3152 excluded.

| Parameter | Truth | Prior | Replicate 3112 | Replicate 3152 | Replicate 3100 | Replicates 3100–3199* |
|---|---|---|---|---|---|---|
| $\delta$ | 1.0 | Unif(0, 100) | 66.41 [42.50, 97.29] | 31.56 [15.15, 48.96] | 1.02 [0.58, 1.57] | 1.20 |
| $\lambda_C$ | 0.07 | Unif(0, 100) | 71.24 [45.08, 98.92] | 39.42 [23.91, 56.54] | 1.51 [$5x10^{-4}$, 3.37] | 1.92 |
| $\lambda_H$ | 49.5 | Unif(0, 100) | 66.03 [51.77, 86.11] | 52.07 [42.40, 62.87] | 48.77 [47.40, 50.32] | 49.54 |
| | | | | | | |
| $\delta$ | 1.0 | Fixed | 1.0 | 1.0 | – | – |
| $\lambda_C$ | 0.07 | Unif(0, 100) | 0.46 [$3 x10^{-4}$, 1.53] | 0.77 [$6 x10^{-3}$, 2.04] | – | – |
| $\lambda_H$ | 49.5 | Unif(0, 100) | 48.20 [46.87, 49.54] | 49.90 [48.35, 51.22] | – | – |

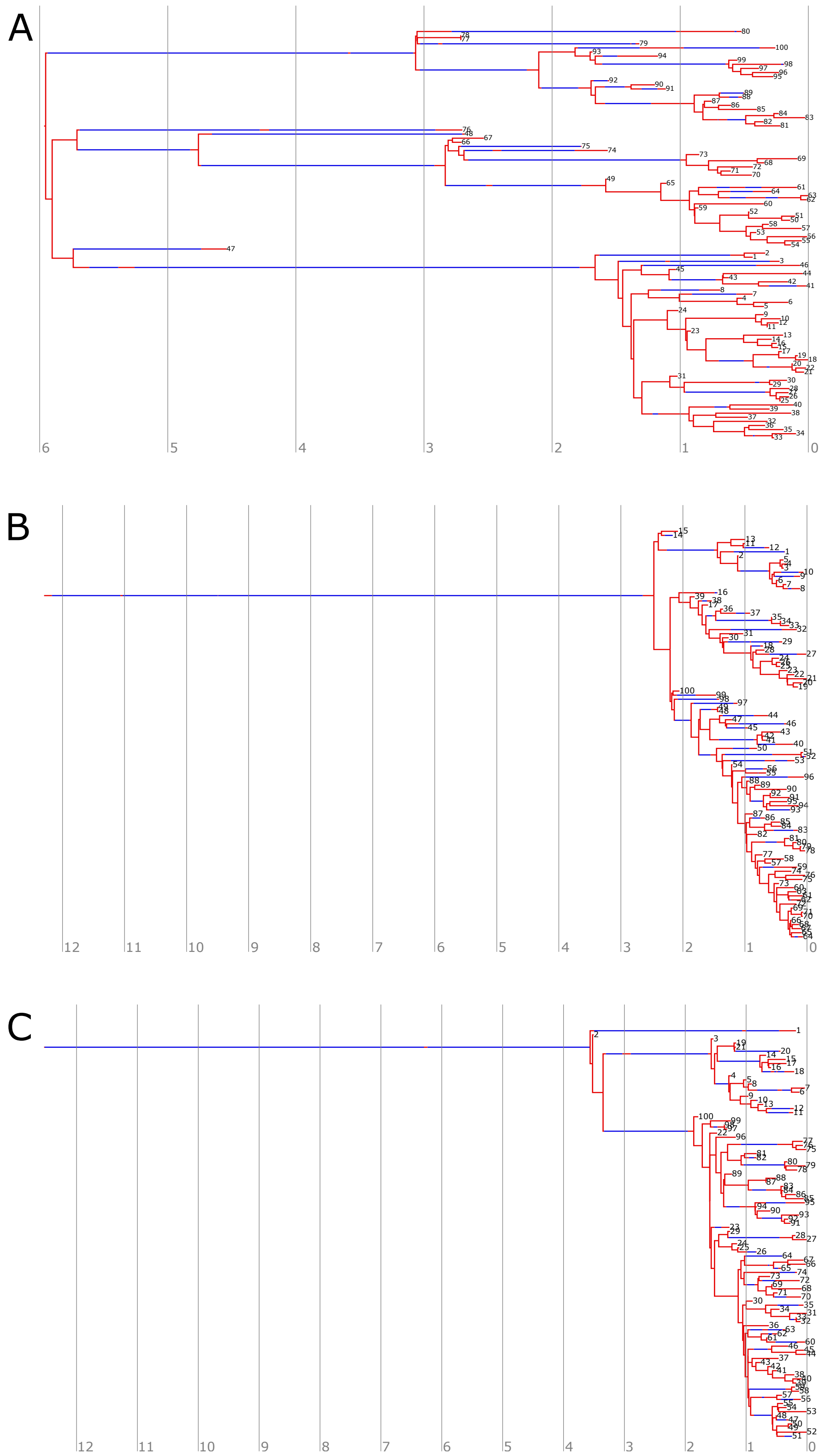


**Figure D.** Simulated sampled transmission trees for simulation replicates 3100 (panel A), 3136 (panel B) and 3189 (panel C) by assuming hospital transmission rate of $\lambda_H = 45.0y^{-1}$ and community sampling rate of $s_C = 0.0001$. For the simulated trees the branches are colored according to the demes: blue color indicates community whereas red color denotes hospital. Transmission tree for simulation replicate 3100 (panel A) represents a case where inferred estimates for the rate to become non-infectious are close to true value ($\delta = 1.0\ y^{-1}$). In all panels, the x-axis represents time, here with the unit being years. Figure created with IcyTree [1].

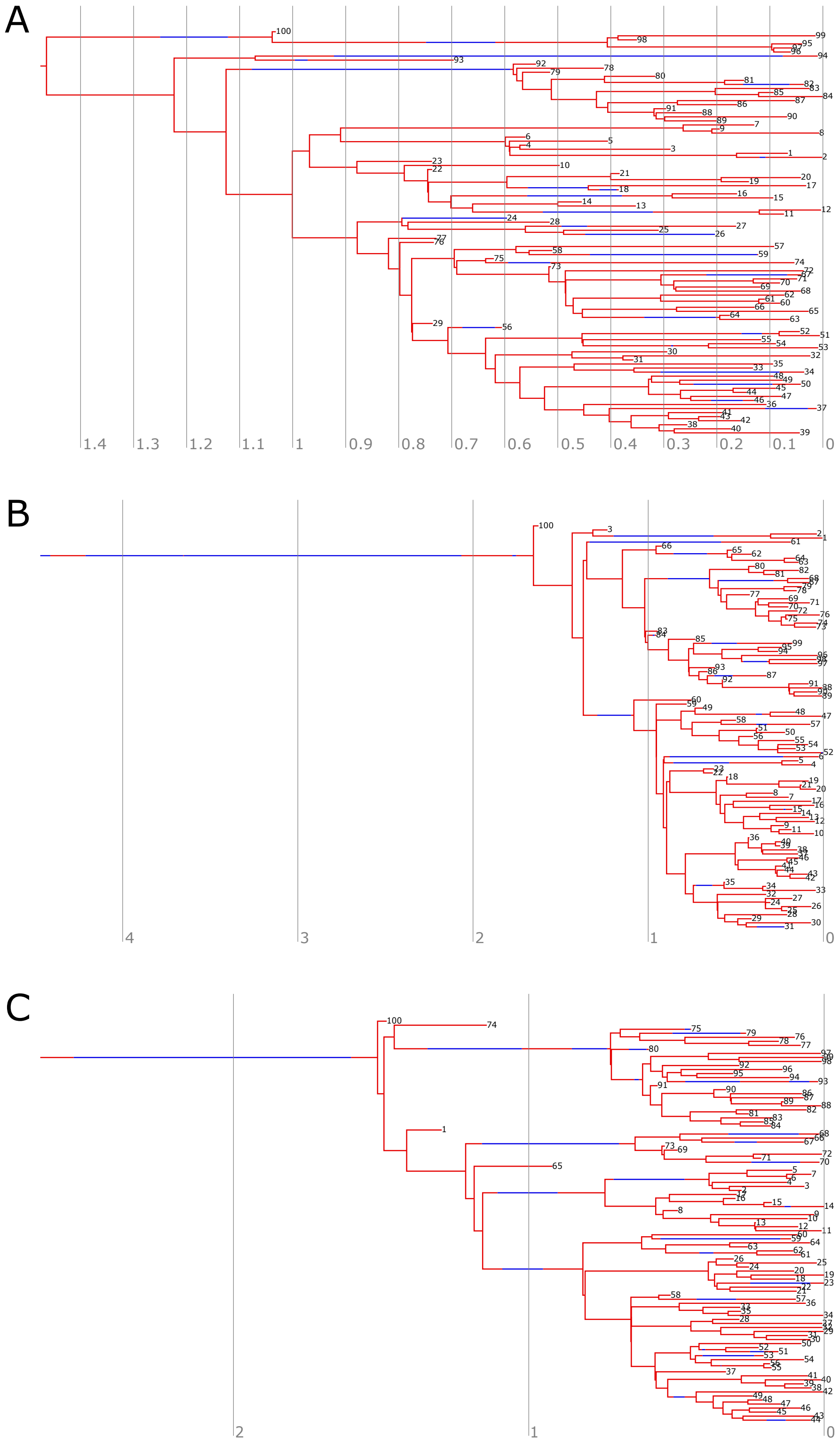


**Figure E.** Simulated sampled transmission trees for simulation replicates 3100 (panel A), 3112 (panel B) and 3152 (panel C) by assuming hospital transmission rate of $\lambda_{\mathrm{H}} = 49.5y^{-1}$ and community sampling proportion of $s_{\mathrm{C}} = 0.0001$. For the simulated trees the branches are colored according to the demes: blue color indicates community whereas red color denotes hospital. Transmission tree for simulation replicate 3100 (panel A) represents a case where inferred estimates for the rate to become non-infectious are close to true value ($\delta = 1.0\ y^{-1}$). In all panels, the x-axis represents time, here with the unit being years. Figure created with IcyTree [1].

## References

[1] Vaughan TG. IcyTree: rapid browser-based visualization for phylogenetic trees and networks. *Bioinformatics*. 2017; 33(15):2392–2394.
